# Ultra-Broadband Microwave Absorption and Programmable Multispectral Camouflage Enabled by Neural-Network-Driven Impedance-Gradient Metadevices

**DOI:** 10.1007/s40820-026-02247-z

**Published:** 2026-06-16

**Authors:** Chen Li, Leilei Liang, Baoshan Zhang, Yi Yang, Guangbin Ji

**Affiliations:** 1 School of Electronic Science and Engineering, Nanjing University, Nanjing, 210093 People’s Republic of China; 2 College of Materials Science and Technology, Nanjing University of Aeronautics and Astronautics, Nanjing, 210016 People’s Republic of China; 3 Department of Chemistry, Tsinghua University, Beijing, 100084 People’s Republic of China

**Keywords:** Microwave absorption, Impedance gradient, Multiscale, Programmable, Radar–infrared–visible camouflage

## Abstract

Achieving omnidirectional multispectral compatible camouflage remains a significant challenge due to the pronounced disparities in electromagnetic wavelengths and constraints on the response mechanisms of natural materials. Herein, neural networks are employed to intelligently optimize the design of multiscale impedance-gradient (IG) metadevices tailored for multispectral compatibility. The macro-gradient unit is engineered with precise impedance matching and high rotational symmetry to provide exceptional microwave ultra-broadband absorption (2–18 GHz), with insensitivity angles reaching 60°. By integrating a polyimide foam substrate with an MXene-functionalized nanostructured photochromic top layer, the finalized device exhibits remarkable infrared thermal insulation (ΔT ≈ 65 °C) and low emissivity (0.38), alongside rapid visible color change (1 ~ 2 s) enabled by nanoscale photochromic switching. Furthermore, IG metadevices deliver programmability and multimodality, alongside impact resistance (~ 30,000 N) and environmental stability. This work provides novel paradigms for the intelligent design of multispectral compatible camouflage systems adaptable to complex scenarios.

**Supplementary Information:**

The online version contains supplementary material available at 10.1007/s40820-026-02247-z.

## Highlights

- An impedance-gradient metadevice for multispectral compatible camouflage was intelligently designed using the neural network.
- The metadevice demonstrates excellent microwave ultra-broadband absorption (2–18 GHz), infrared thermal insulation (ΔT≈65 °C), low emissivity (0.38), and rapid visible color change (1–2 s).
- The assembled device offers programmability and multimodality while also exhibiting impact resistance (~30,000 N) and environmental stability.

**Supplementary Information:**

The online version contains supplementary material available at 10.1007/s40820-026-02247-z.

## Graphical abstract

**Figure:**
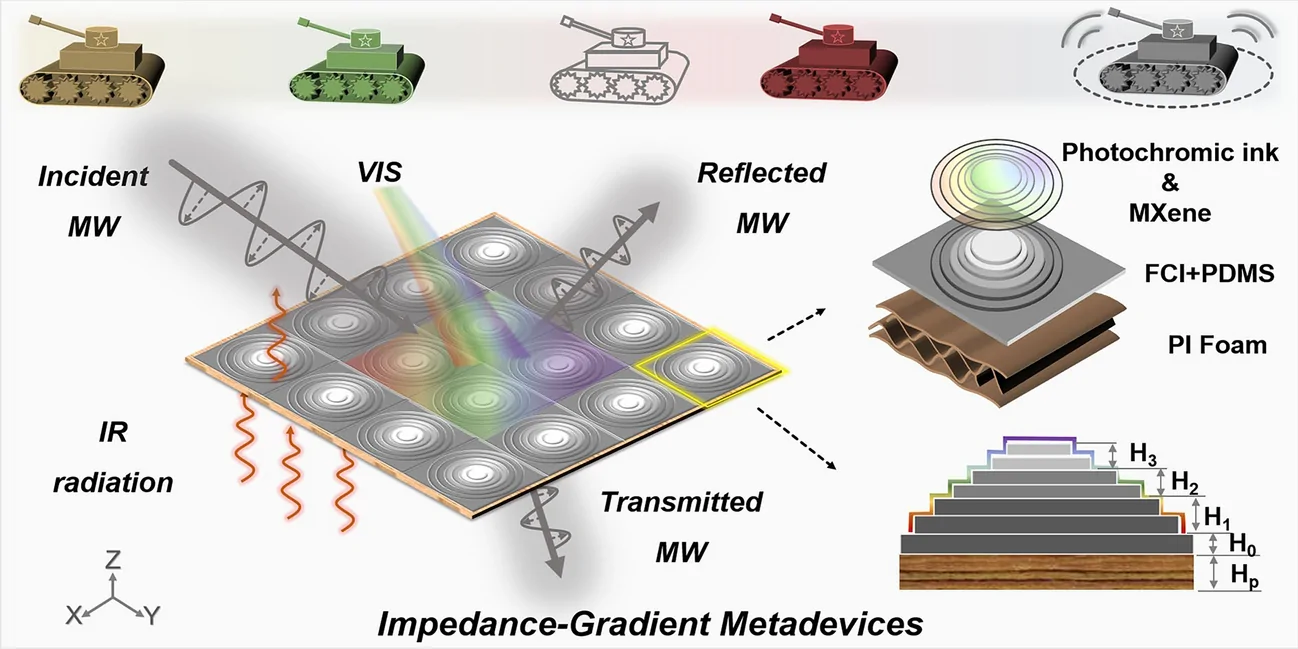

## Introduction

Camouflage serves as a pivotal technology for weaponry and equipment to evade threats posed by enemy reconnaissance methods [1–3]. Nowadays, significant progress has been made in anti-detection defense strategies for different electromagnetic (EM) spectrum bands (such as radar, terahertz, laser, infrared (IR), and visible (VIS)) [4–7]. However, single-band camouflage technology proves ineffective against multispectral cooperative detection, necessitating urgent development of compatible camouflage solutions that span EM wavelengths [8, 9]. The emergence of advanced precision-guided weapons, especially the "Destroy immediately upon discovery" doctrine, imposes unprecedented challenges for camouflage technology [10]. Especially for ground targets striving to survive in complex and harsh environments, the development of radar-IR-VIS multispectral compatible systems for authentic concealment and deceptive display is of paramount importance [11, 12]. Given the mutually constraining EM response mechanisms, functional integration, and thermal management limitations across different bands, achieving compatible camouflage presents formidable challenges. Radar camouflage primarily emphasizes high absorption within the 2–18 GHz range to minimize radar cross section (RCS) signatures [13, 14]. IR camouflage focuses on modulating temperature and emissivity to suppress target radiation intensity [15–17]. VIS camouflage hinges on adjusting color and spectral characteristics to reduce contrast against the background [18, 19]. Researchers have effectively integrated VIS-IR stealth devices through metasurface designs, demonstrating exceptional color matching and emissivity control [12, 16]. Additionally, leveraging material properties and multilayer structures to manipulate surface reflectivity enables radar stealth and IR thermal camouflage [20, 21]. Although progress has been made in dual-band compatible stealth, two critical challenges persist in the exploration of radar-IR-VIS multispectral compatibility. The narrow microwave absorption (MWA) bandwidth caused by matching mechanisms and the contradictory coordination of cross-wavelength thermal management.

Broadband absorption is typically achieved through the synergistic optimization of magneto-electric composites, multilayer/hierarchical structure, or incentive-driven frequency shift designs [22–26]. Currently, these strategies can yield fairly impressive MWA results. Unfortunately, the intrinsic incompatibility between the EM wavelength and the material’s inherent response has become a bottleneck for ultra-broadband MWA technology. Considering impedance-matching mechanisms, gradient structures can effectively facilitate a smooth transition from air to its characteristic impedance, thereby substantially expanding the bandwidth [27, 28]. Extensive research has demonstrated that metamaterials [29, 30], multilayer composite aerogels [31, 32], and films [33, 34] with gradient structures exhibit exceptional broadband MWA. Wherein metastructure design enables the construction of structural–functional devices, facilitating cross-wavelength integration while simultaneously achieving coordinated stealth and thermal management [35]. However, determining the optimal structural parameters for these designs has traditionally required extensive experimental iterations, rendering the fabrication process cumbersome and inefficient. To mitigate temporal and economic costs, a neural network (NN)-based predictive approach from the field of artificial intelligence can be employed for parameter optimization. Currently, this strategy is widely applied in prominent domains such as medical diagnostics [36, 37], energy and environmental data collection [38, 39], and semiconductor device manufacturing [40, 41]. NNs not only autonomously extract spatial features to model complex relationships but also offer high computational efficiency and accuracy, making them promising for guiding the development of compatible stealth devices. Recent studies on NN-assisted intelligent metasurface designs for precise EMW manipulation further validate the reliability of this predictive methodology [14, 42]. Therefore, NN-guided material–structure-device integration offers a transformative approach to overcoming existing limitations for achieving multispectral compatible stealth.

**Fig. 1:**
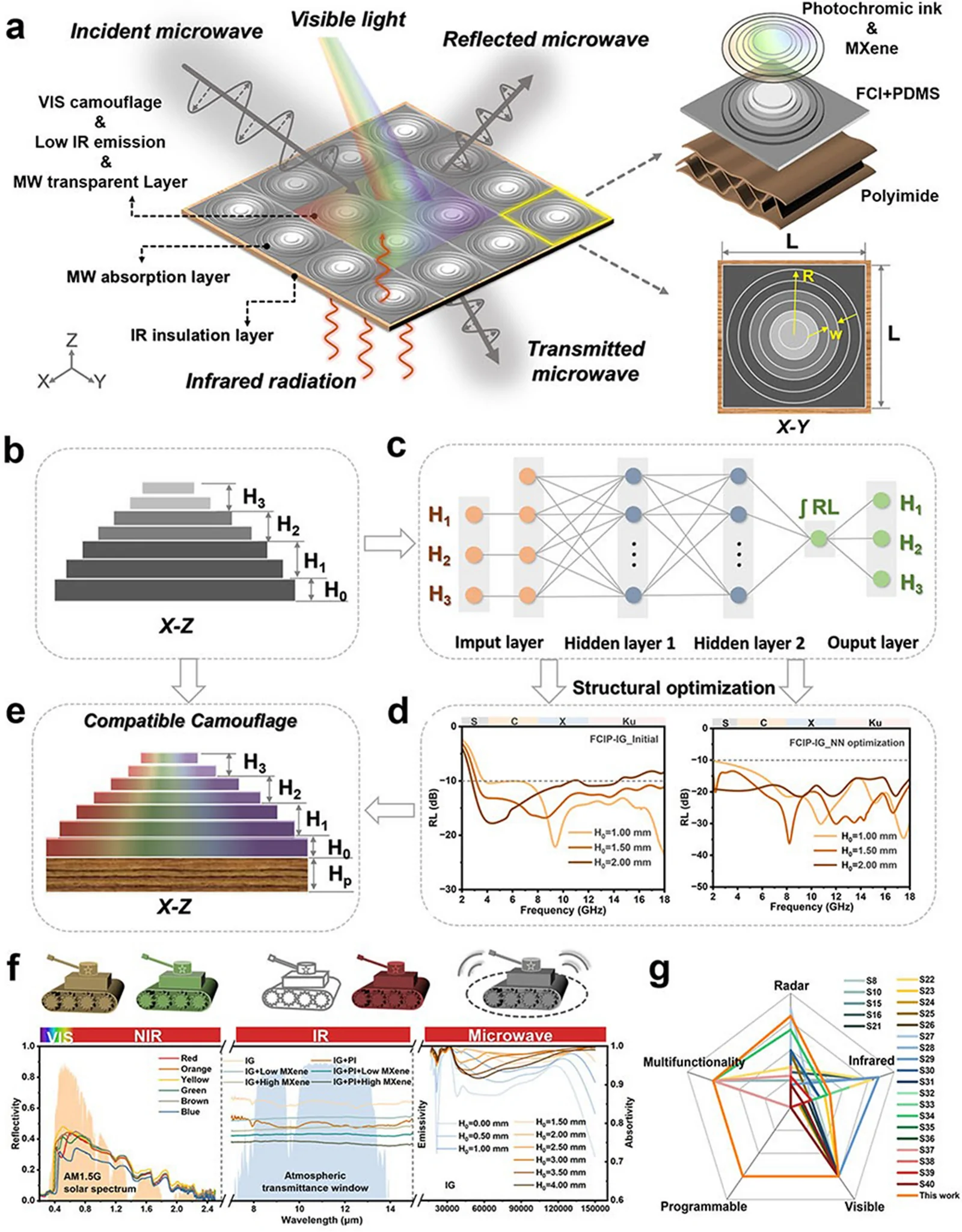
Multispectral compatible camouflage device design. **a** Schematic diagram of periodic IG multispectral compatible camouflage device. **b** Structural parameters of the device. **c** Schematic diagram of NN model (input layer, hidden layer, output layer). **d** Reflectance curves of IG metadevices before and after NN optimization. **e** Schematic diagram of radar-IR-VIS compatible camouflage application. **f** Reflectivity/emissivity/absorptivity spectra (from VIS to MW). **g** Performance of the IG metadevice

Herein, we present a programmable impedance-gradient (IG) metadevice for multispectral compatible camouflage (Fig. 1a). The top layer, composed of nanostructured photochromic ink and MXene, serves as a VIS camouflage and low IR emissivity layer while simultaneously permitting microwave transmission. The middle part consists primarily of multiple radar resonant units, constructed by blending flake carbonyl iron particles (FCIP) with a flexible polydimethylsiloxane (PDMS). The bottom is equipped with a polyimide (PI) thermal insulation foam layer, designed to block IR radiation energy. Benefiting from impedance matching and symmetric structural units, the integrated device achieves ultra-broadband absorption (2–18 GHz) and incidence angle insensitivity with the aid of NN optimization. The synergistic effect between PI foam and low-emissivity MXene achieves comprehensive concealment of the target substrate, with FCIP also playing a crucial role. In the face of ever-changing operational environments, weaponry can be seamlessly concealed within various background colors. Furthermore, the independent operation of each unit allows for VIS-IR information encoding and decoding, thereby possessing multimodal characteristics. The robust impact resistance, self-cleaning, anti-icing, and chemical stability of the compatible stealth device lay a solid foundation for long-term application in extremely harsh environments.

## Experimental Section

### Preparation of Flexible FCIP/PDMS

Different masses of FCIP were incorporated into a viscous polydimethylsiloxane (PDMS) matrix along with a curing agent, followed by mechanical homogenization for 30 min using an electric mixer to obtain uniformly dispersed FCIP/PDMS. The mass ratios of FCIP to PDMS were maintained at 1:2, 1:1, and 2:1, respectively.

### Preparation of Photochromic Films

Various colored (red, orange, yellow, green, blue, and brown) 5 g photochromic inks were uniformly blended with MXene solutions of both low (5 mg mL^−1^) and high (20 mg mL^−1^) concentrations through mechanical stirring.

### Preparation of Impedance-Gradient Metadevice

To fabricate the IG metadevice, a layered casting process was employed. First, FCIP/PDMS-12 was poured into a prefabricated polytetrafluoroethylene mold to create the two flexible absorbing layers of the top layer. This was followed by a drying step in an oven at 60 °C for 40 min. Subsequently, FCIP/PDMS-11 was poured into the same mold, forming the two flexible absorbing layers of the middle layer. A second drying step at 60 °C for 60 min was then performed. Finally, FCIP/PDMS-21 was poured into the mold, creating the two flexible absorbing layers of the bottom layer. After complete drying, the IG metadevice was carefully demolded.

### Construction of Periodic IG Arrays

The IG units were arranged in a 4 × 4 matrix with a horizontal distance of 45 mm between the central axes of adjacent units. This array was then transferred onto rectangular (180 mm × 180 mm) flexible FCIP/PDMS-21 films, utilizing a thin layer of PDMS as an adhesive. Subsequently, PI thermal insulation foam layers of varying thicknesses were attached. Finally, pre-homogenized photochromic ink was coated onto the upper surface of the periodic IG structure. The assembled device measured 180 mm in both length and width, with a total height ranging from 8 to 18 mm.

### Characterization

The phase composition of FCIP was analyzed using X-ray diffraction (XRD, Smartlab 9), Fourier-transform infrared spectroscopy (FTIR, Nicolet-Is10), and X-ray photoelectron spectroscopy (XPS, Escalab 250Xi). The microstructure and morphology of both FCIP and FCIP/PDMS films were characterized via scanning electron microscopy (SEM, Apreo 2) and transmission electron microscopy (TEM, Talos F200X). Ultraviolet–visible–near-infrared (UV–VIS-NIR) spectrophotometry (Uv3600IPLUS) was employed to measure the absorption and reflection spectra of the FCIP and photochromic films within the 200–2500 nm range. Hydrophilic and hydrophobic properties were evaluated by contact angle measurements (Theta Flex), while magnetic properties were investigated utilizing a vibrating sample magnetometer (VSM, MPMS). A drop-weight impact tester (Instron 9450) was used to collect impact data for evaluating impact resistance and mechanical performance. A smartphone camera (Vivo X100, China) was used for structural imaging and video recording. Real-time infrared (IR) images and videos were captured using an IR thermal imager (Fotric 227 s). Dynamic IR emissivity spectra were determined using a Fourier-transform IR spectrometer (FTIR, Tensor 27) coupled with a blackbody radiation source (DY-HT1, D-MEI Instruments). Microwave reflectivity was assessed via the NRL-arc method, employing a vector network analyzer (VNA, Agilent E8363B).

## Results and Discussion

### Multispectral Compatible Camouflage Metadevices

Periodically (4 × 4) impedance-gradient (IG) metadevices exhibit a highly symmetrical, stepped architecture (Fig. 1a, b). At the heart of the metadevice lies a central gradient radar resonant unit, primarily fabricated with the aid of custom-designed molds (Fig. S1). The detailed fabrication procedures and materials characterization are provided in Note S1 and Figs. S1–S7. The initial structural parameters of the metadevice (L = 45 mm, R = 20 mm, and w = 2 mm) were optimized through electromagnetic simulation and selected as optimal values, remaining fixed. Modulation of the MWA performance can be achieved by adjusting the H_0_, H_1_, H_2_, and H_3_ parameters. While the possible combinations of H₀-H₃ are virtually infinite, an initial reference set (1, 2, 2, and 2 mm) is provided for comparative analysis. To expedite structural optimization, NN was utilized to predict the optimal parameter combination. With H_0_ fixed at 1 mm, the *RL-f* curve dataset pertaining to H_1_, H_2_, and H_3_ was employed for training, yielding the maximum integrated value (< −10 dB) of the absorption curve along with the corresponding three thickness parameters (Fig. 1c). Comparative results demonstrate that the optimized device achieves an effective absorption bandwidth (EAB) spanning 2–18 GHz (Fig. 1d). Furthermore, by adjusting the thickness (Hₚ) of the bottom thermal-insulating PI foam (1, 3, 5, and 7 mm), thermal radiation was effectively blocked, enabling successful IR camouflage. Meanwhile, the integration of different photochromic inks (such as red, orange, yellow, green, brown, and blue) atop the structure facilitated adaptive concealment across diverse environments (Fig. 1e). Compared to other systems, the integrated impedance-gradient components demonstrate VIS color change, low IR emissivity, and high MWA performance, while concurrently offering programmability and multifunctionality (Fig. 1f, g).

### Microwave Absorption Performance

**Fig. 2:**
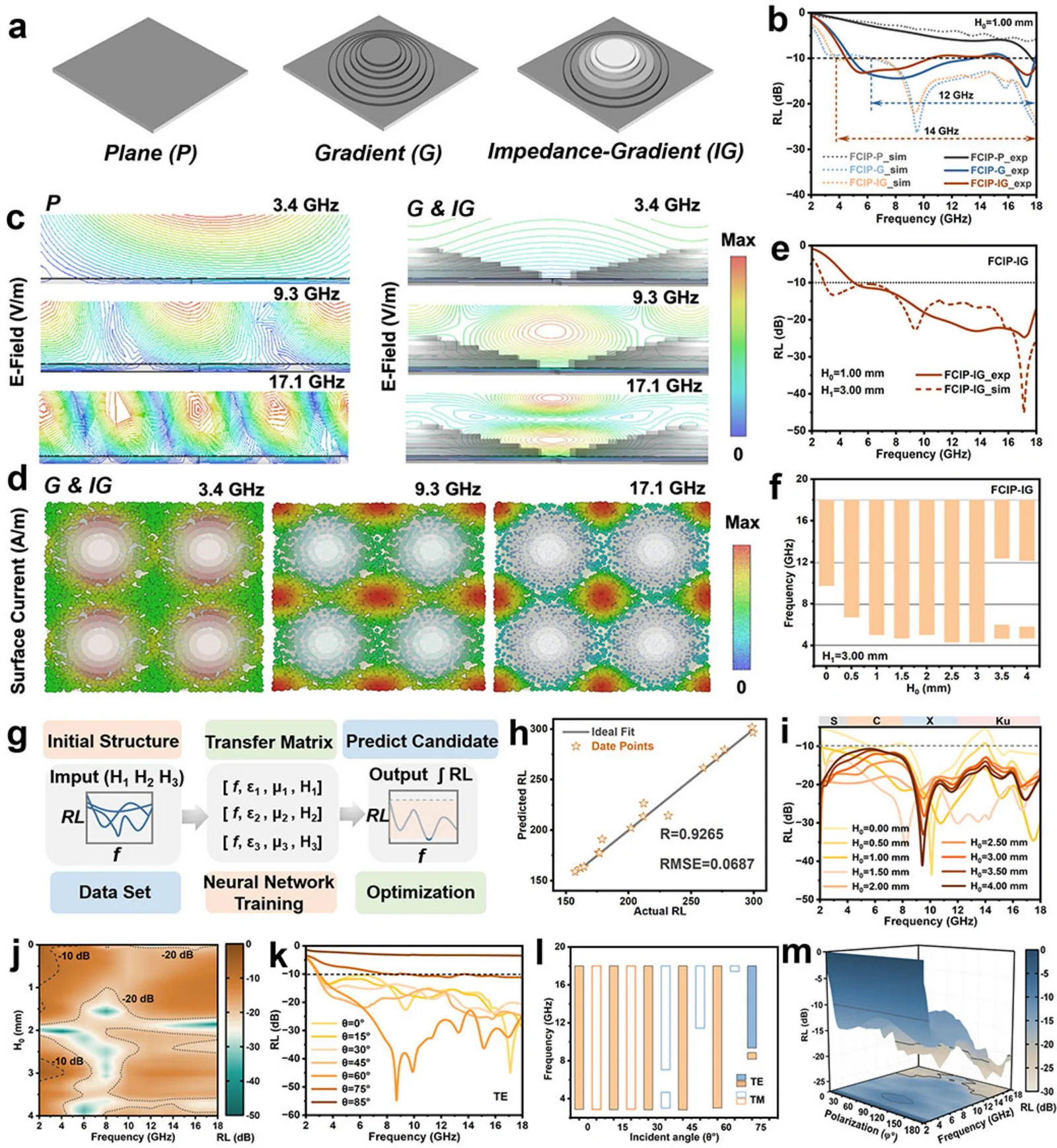
Microwave absorption performance. **a** Schematic diagram of planar (P), gradient (G), and impedance-gradient (IG) structures. **b** Comparative analysis of simulated and experimental *RL-f* curves for P, G, and IG devices (H_0_ = 1.00 mm). **c** Electric field of P and IG devices. **d** Surface current distribution of IG devices. **e** Comparative analysis of simulated and experimental *RL-f* curves for IG devices (H_0_ = 1.00 mm, H_1_ = 3.00 mm). **f** Bandwidth statistical diagram of IG devices under different H_0_ (H_1_ = 3.00 mm). **g** Flowchart of NN prediction. **h** Comparison of predicted values and actual values after NN training. **i** *RL-f* curves under different H_0_ after NN parameter optimization. **j** 2D absorption color map after NN parameter optimization. **k** *RL-f* curves in TE modes at different θ. **l** Statistical diagram of EAB at different θ in TE and TM modes. **m** 3D absorption color map of different φ in TE mode

The designed IG structure is compared with planar (P) and gradient (G) (Fig. 2a). FCIP/PDMS-21 was selected for both P and G structures due to its superior MWA performance characteristics (Figs. S8–S11, Notes S2 and S3). The NRL-arc method was employed to measure the reflectivity of the device, (Fig. S12). Figure 2b presents the CST-simulated theoretical and experimentally measured *RL*-*f* curves for FCIP-P, FCIP-G, and the FCIP-IG structure at H₀ = 1 mm. Owing to the ingenious impedance-matching design, the EAB exceeds 10 GHz. To quantitatively verify the impedance-matching performance, the normalized input impedance Z_in_​/Z_0_​ of the P, G, and IG structures is calculated across the 2–18 GHz band using S-parameter data derived from Smith charts (Fig. S13). The stable impedance trajectory of the IG structure confirms its superior and smooth impedance matching to free space, which is responsible for the broadband absorption behavior. Adjusting H₀ yields distinct *RL-f* curves, demonstrating that greater bottom-layer thickness enhances low-frequency absorption. This trend was confirmed across FCIP-P, FCIP-G, and the FCIP-IG structure (Fig. S14). To elucidate the underlying MW dissipation mechanism, we performed a comparative simulation analysis of the P and IG devices. The distributions of the electric field (E) and magnetic field (H) were monitored at representative *RL-f* peaks (3.4, 9.3, and 17.1 GHz). For P devices, narrow absorption bandwidth is observed as the absorber thickness approaches λ/4, which only excites a quarter-wavelength resonance at a specific frequency. In contrast, the IG device integrates resonances from adjacent frequencies, resulting in an ultra-broadband response. Importantly, as the frequency increases, strong coupling between adjacent IG units creates localized "hot spots" (Figs. 2c and S15a). Consequently, the loss mechanism involves multiple quarter-wavelength resonances at low frequencies and diffraction effects arising from the structural interactions at high frequencies [43, 44]. Surface current (${J}_{s}$) predominantly concentrates along the edges of the periodic units, indicating field-enhanced ohmic losses (${P}_{s}={R}_{s}{\left|{J}_{s}\right|}^{2}$) (Figs. 2d and S15b). The power flow (*S*) reveals the energy transmission pathways, with high-order mode diffraction effect occurring between two IG units. This effect causes a portion of the EMWs to alter their propagation direction and enter the absorber through the structural edges (Fig. S16). Furthermore, the multi-order dipole resonances generated by the periodic structure confine energy between neighboring units, manifesting as enhanced surface power loss density (Fig. S17). The trapped MW energy is subsequently dissipated by the IG structure, achieving the goal of high absorption efficiency.

Improved low-frequency absorption and weakened high-frequency absorption in Fig. S14 suggest that simply increasing H_0_ is insufficient for full-band absorption. This work simultaneously modulated H_1_, H_2_, and H_3_ of the IG structure, with priority given to adjusting H_1_. When H_1_ was increased to 3 mm, both low and high-frequency absorption were maintained below −10 dB, resulting in a bandwidth of 4–18 GHz (Fig. 2e). By tuning H_0_ from 0 to 4 mm, the corresponding *RL-f* curves revealed that the EAB could nearly reach 14 GHz (Figs. 2f and S18). Due to the limited sample size and edge effects during actual testing, there are slight discrepancies between the results and the simulation predictions. These results demonstrate that modulating H_1_–H_2_ offers remarkable efficacy, yet substantial challenges remain in achieving such simultaneous regulation. NNs effectively address this problem by enabling rapid prediction of optimal parameter combinations. The original dataset was constructed from a series of *RL-f* curves via CST, obtained by varying H_1_–H_2_ (Fig. S19). The input features consist of the adjustable thickness parameters [H_1_​, H_2_​, H_3​_], with a step size set to 0.01. The parameter vectors [f, ${\varepsilon}_{1}$, ${\upmu}_{1}$, H₁], [f, ${\varepsilon}_{2}$, ${\upmu}_{2}$, H_2_], and [f, ${\varepsilon}_{3}$, ${\upmu}_{3}$, H_3_] are used in the transfer matrix method (TMM) to calculate the *RL* integral values for dataset construction. The NN consists of two hidden layers, each containing ten neurons. The output layer presents the optimal integrated values (*RL* < -−10 dB) across 2–18 GHz along with their corresponding [H_1_, H_2_, H_3_] (Figs. 2g and S20). The double-hidden-layer neural network is rationally chosen for IG metadevices optimization. It offers superior efficiency and accuracy over both traditional machine learning and advanced deep models. The root means square error (RMSE) of 0.0687 and correlation coefficient (R) of 0.9265 verify its high prediction reliability (Fig. 2h and Table S1). SHAP analysis is used to address the black-box limitation (Fig S21). After training, the predicted maximum *RL* integral is 319.07, with H_1_, H_2_, and H_3_ being 5.00, 3.84, and 2.93 mm, respectively. On this basis, by adjusting H_0_ within 0–4 mm, the *RL-f* curves and 2D absorption maps indicate that the EAB can fully cover the 2–18 GHz (Fig. 2i, j). It is worth emphasizing that the thin surface layer of MXene/photochromic ink on the IG unit permits MWs to pass through without any adverse effect (Fig. S22). The thickness of the bottom PI foam layer should be controlled, as excessive thickness would compromise the overall MWA performance (Fig. S23). Additionally, a rotationally symmetric cylindrical design was deliberately adopted to eliminate directional dependence to ensure stable MWA performance of the stealth device across wide incidence angles (θ) and arbitrary polarization angles (φ) (Fig. S24a). For both transverse electric (TE) and transverse magnetic (TM) polarization modes under varying incidence angles, the EAB nearly covers the entire 2–18 GHz when θ<30°. Notably, for TE mode, the EAB remains fully intact even at a 60° incidence angle (Figs. 2k, l and S24b). Meanwhile, its rotational symmetry ensures remarkable polarization angle insensitivity (Figs. 2m and S25). Thus, the IG device achieves broadband, omnidirectional radar stealth through symmetrical configurations, impedance gradation, and resonant effects.

### Infrared Stealth Performance

**Fig. 3:**
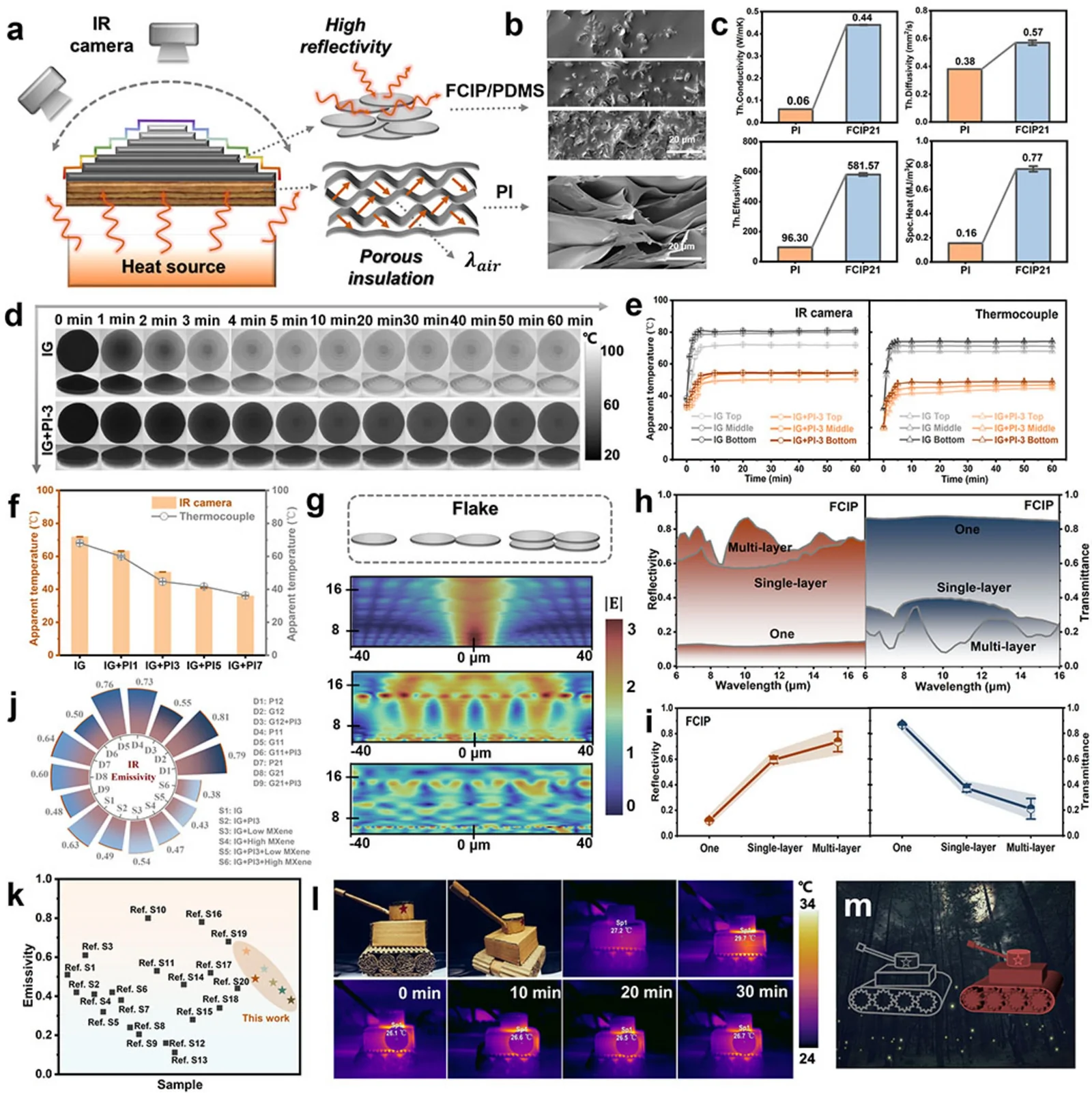
Infrared stealth performance. **a** Schematic diagram of IR stealth mechanism. **b** Cross-sectional SEM images of FCIP/PDMS and PI. **c** Thermal conductivity, heat storage coefficient, thermal diffusivity, and specific heat of PI and FCIP/PDMS. **d** Thermal IR images of IG and IG + PI-3 within 0–60 min. **e** Top, middle, and bottom layers apparent temperature curves of IG and IG + PI-3 under IR camera and thermocouple. **f** Apparent temperature (60 min) of IG, IG + PI-1, IG + PI-3, IG + PI-5, and IG + PI-7 under IR camera and thermocouple. **g** Electric field distribution, **h** Reflectivity and Transmittance (8–14 μm) of one, single-layer, and multilayer FCIP under optical simulation (FDTD). **i** Statistical of average reflectivity and transmittance values. **j** Statistical graph of IR emissivity. **k** Comparison of IR emissivity with other materials. **l, m** Schematic diagram of IR thermal camouflage for tanks

The porous PI foam acts as a barrier, impeding the outward transfer of target heat [45, 46]. The FCIP and surface MXene reflect ambient IR radiation, thereby diminishing the metadevice’s thermal signature (Fig. 3a, b). The PI with its exceptionally low thermal conductivity (0.06 W mK^−1^), high heat storage coefficient (96.30), and low thermal diffusivity (0.38 mm^2^ s^−1^), provides robust thermal insulation (Fig. 3c). Meanwhile, the FCIP/PDMS layer achieves "thermal synchronization" with the environment thanks to its suitable thermal conductivity and heat storage capacity. This prevents abnormal temperature rises that would result from complete insulation [47, 48]. The IG structure placed on a 100 °C heating stage was monitored simultaneously by an IR camera and a thermocouple (Fig. S26). The thermal insulation effect varied with different PI layer thicknesses (1, 3, 5, and 7 mm), and the apparent temperature changes of the FCIP/PDMS-21, FCIP/PDMS-11, and FCIP/PDMS-12 layers within the gradient structure also differed (Videos S1 and S2). Taking the IG + PI-3 as a representative example, the apparent temperature variation over 0–60 min was recorded. Compared to the IG without a PI layer, the apparent temperature of IG + PI-3 stabilized at around 50 °C, indicating long-term stability (Fig. 3d, e). Simultaneously, the temperature measured by the thermocouple closely matched the radiative temperature detected by the IR camera, confirming that the addition of the PI layer effectively reduced the temperature difference between the target and the background (Fig. 3f). Due to the influence of the gradient structure, the apparent temperature of the top layer was slightly lower than that of the bottom layer. Furthermore, as the PI thickness increased, the apparent temperature gradually decreased, with temperature differences (∆T) for 1, 3, 5, and 7 mm reaching approximately 40, 50, 60, and 65 °C, respectively (Figs. 3d, e and S27–S29).

The interaction mechanism between FCIP and IR waves was investigated via FDTD simulations (Fig. S30). The electric field, magnetic field, and power distribution of one, single-layer and multilayer FCIP were analyzed across the 8–14 μm. With increasing FCIP, the filler particle size exceeds the IR wavelength, causing intense light reflection based on geometric optics principles (Figs. 3g and S31). The reflectance and transmittance curves show that the multilayer FCIP has high reflectance and low transmittance (Fig. 3h). Statistical analysis in Fig. 3i reveals an average reflectance exceeding 80% within the 8–14 μm. To validate the simulation results, IR emissivity tests were conducted to assess the practical influence of FCIP and MXene on IR camouflage. The FCIP-P exhibits an emissivity of approximately 0.7, while the FCIP-IG reduces it to ~ 0.6. MXene achieves an ultra-low infrared emissivity through the synergistic effects of conductive network reflection, interlayer thermal barrier effect, and interfacial polarization relaxation. By precisely controlling its concentration, the trade-off between low infrared emissivity and high microwave transmittance can be effectively balanced. Therefore, the incorporation of an MXene surface layer further suppresses emissivity to 0.5. By fine-tuning the MXene concentration in the photochromic ink, the emissivity can be stabilized at 0.38, and this emissivity reduction is independent of the photochromic component itself (Figs. 3j and S32). In summary, the designed IG structure combines robust thermal insulation and low IR emissivity, performing as well as or better than previously reported structural materials (Fig. 3k and Table S2). In a practical demonstration, a tank model was used as an IR camouflage target. IG devices were strategically placed over heat-prone areas, allowing the tank to blend seamlessly into its surroundings and maintain stable concealment (Fig. 3l, m).

### Visible Camouflage Performance

**Fig. 4:**
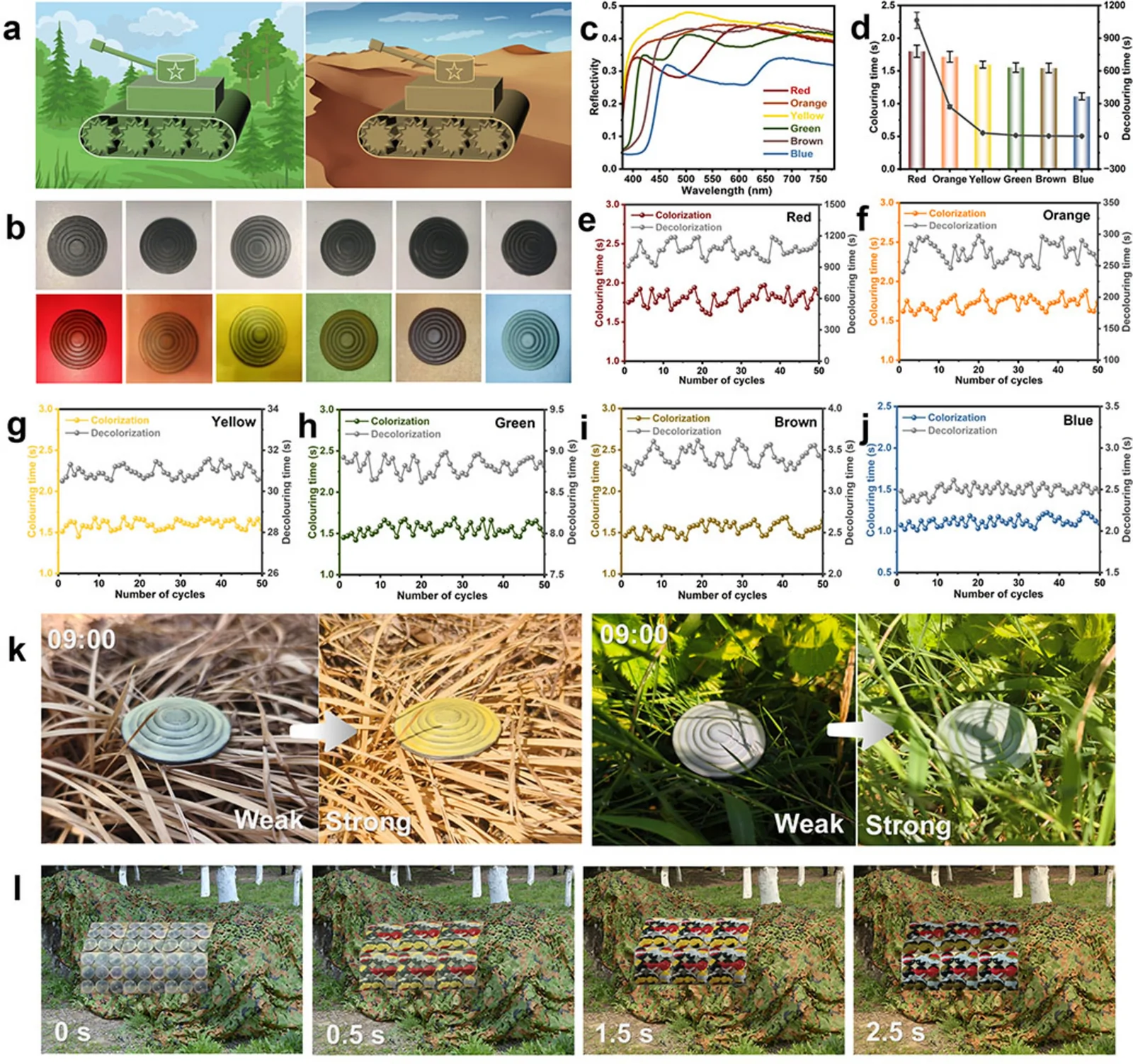
Visible camouflage performance. **a** Schematic diagram of VIS camouflage for forest and desert warfare. **b** Comparison of color-changing effects of different photochromic pigments. **c** Reflectivity (380–780 nm) of different photochromic pigments. **d** Statistics on the coloring and decoloring times of different photochromic pigments. Coloring and decoloring time of **e** red, **f** orange, **g** yellow, **h** green, **i** brown, and **j** blue photochromic ink within 50 cycles. **k** Schematic diagram showing the color change of the metadevice under weak and strong light illumination on a grass background. **l** Schematic diagram of VIS camouflage pattern design in actual scenarios

To blend with complex work environments (*e.g.*, forest, desert), photochromic inks are selected. Their chemical structure undergoes dynamic, reversible changes in response to light exposure (Fig. 4a) [49]. The photochromic mechanism is based on the reversible ring-opening/ring-closing isomerization of pyran/oxazine rings in naphthopyran and spirooxazine derivatives. Coloration occurs under visible-light irradiation via C–O bond cleavage and π-conjugation extension, whereas fading takes place in the dark through reverse isomerization. The IG device, coated with diverse photochromic layers (*e.g.*, red, orange, yellow, green, brown, and blue), exhibits remarkable chromatic similarity to the background after transformation (Figs. 4b and S33). UV–VIS-IR spectroscopy revealed a visible reflectance value of ~ 40% (Figs. 4c and S34a). The CIE 1931 chromaticity diagram and empirical measurements were employed to validate color deviation [50]. The rapid light-induced chemical response of the photochromic inks did not cause a significant change in chromatic coordinates because they faded quickly when shielded from light (Fig. S34b). We monitored the coloring and decoloring time of different photochromic films over 50 cycles, with yellow, green, brown, and blue demonstrating superior VIS color change performance (Fig. 4d−j and Video S3). Films incorporating low-energy-barrier photochromic molecules exhibited rapid coloration (~ 1.1 s) and fading (~ 2.5 s), enabling swift adaptation to different scenarios. Moreover, the response time remained stable over 50 cycles, demonstrating the long service life of the photochromic films. In demonstrations, the IG device displayed the expected VIS camouflage effect when placed on yellow and green grassland under both low and strong illumination (Fig. 4k). Across different times of day, the monitored color change indicates that light intensity from 09:00 to 14:00 produces a more pronounced camouflage effect (Fig. S35 and Table S3). For complex environments, camouflage patterns can be designed to visually disrupt detection. The IG devices exhibited color changes under varying light intensities, readily deceiving opponents (Fig. 4l).

### Programmable and Multimodal Design

**Fig. 5:**
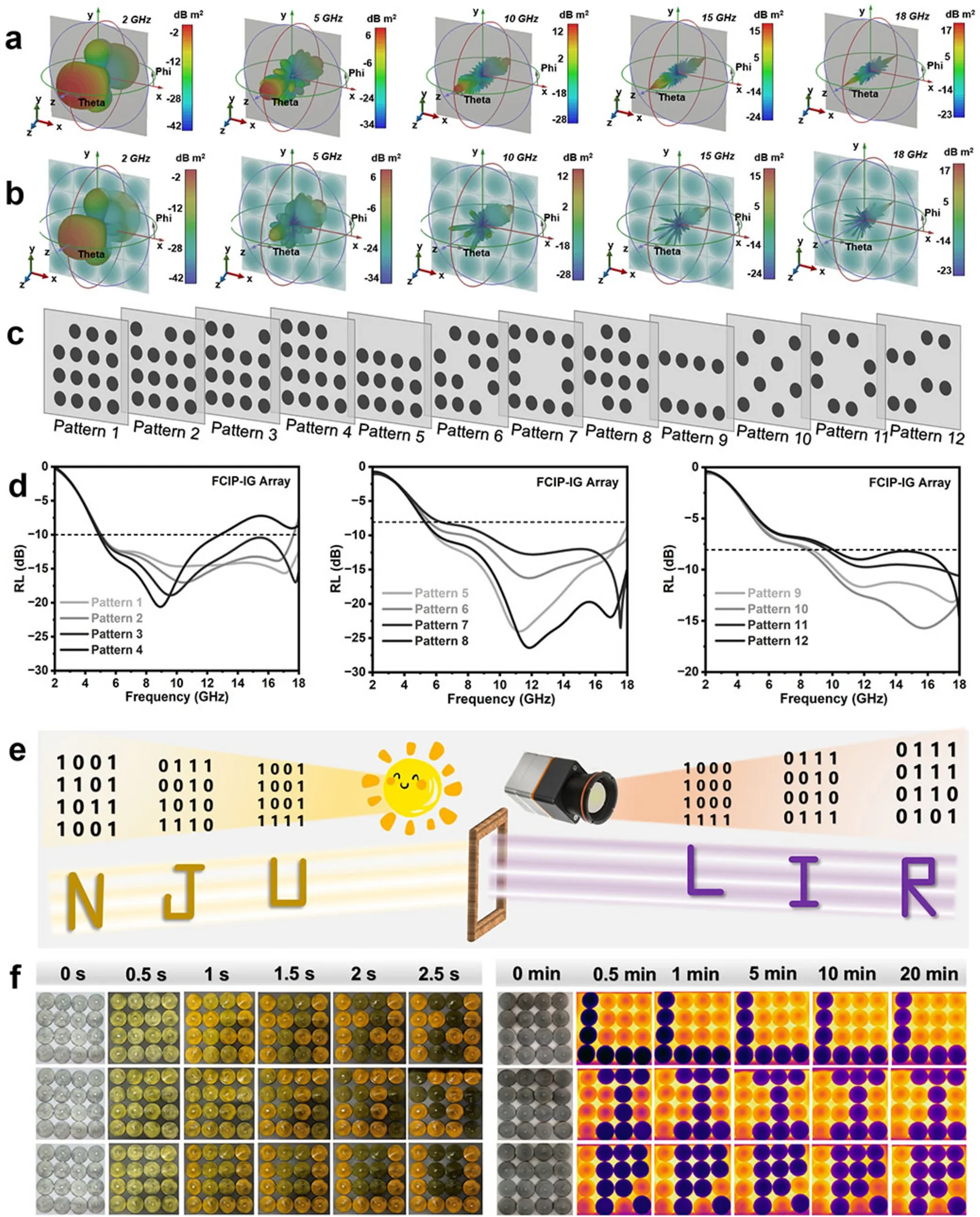
Programmable and multimodal design. Schematic illustration of radar scattering signals for **a** PEC and **b** IG devices (2 GHz, 5 GHz, 10 GHz, 15 GHz, and 18 GHz). **c** Schematic diagram of 12 pattern coding designs. **d** *RL-f* curves for 12 patterns. **e** Schematic diagram of switching between VIS and IR encoding/decoding modes. **f** “NJU” displayed in VIS mode hides the “LIR” displayed in IR mode

The array consists of mutually independent unit structures, with a 4 × 4 matrix employing binary coding "0" and "1." For static radar coding, "0" represents a P structure, while "1" denotes an IG structure. The static radar coding exhibits high coding accuracy, stable electromagnetic response, and excellent anti-interference capability. By utilizing different coding schemes, false scattering centers can be generated to create radar illusions, simulate the signatures of other targets, or embed specific patterns for friendly radar identification [51]. First, radar cross section (RCS) simulations were conducted for perfect electric conductor (PEC), P structures, and IG structures (45 mm x 45 mm), monitoring RCS reduction at 2, 5, 10, 15, and 18 GHz (Fig. S36a–c). Three-dimensional RCS diagrams revealed that the IG structure significantly reduces radiation dimensions, enhancing MWA performance (Fig. S37). Two-dimensional curves spanning varying angles and wavelengths demonstrated reduced RCS values across nearly the entire −90° to 90° range. Statistical analysis at θ=0° showed the RCS_max_ of −41.17 dB m^2^, achieved by the IG structure at 15 GHz (Fig. S38). Upon assembling the IG structures into a 4 × 4 array (180 mm×180 mm), RCS calculations indicated reductions of 2.92, 12.24, 15.94, 23.38, and 34.89 dB m^2^ at different frequencies compared to pure PEC (Figs. 5a, b and S36d, e). Representative examples where one "1" was replaced with "0" exhibited minimal changes in radar scattering signals (Figs. S39–S41). Further substitutions of four and eight "1" units with "0" resulted in slightly enhanced signals, yet the performance remained sufficient to evade enemy detection (Figs. 5c, S42–S45 and Table S4).

Reflectivity tests were conducted for these 12 coding designs. Patterns 1–4 exhibited a low-frequency shift in absorption curves while maintaining near-perfect absorption (< -−10 dB) across 5–18 GHz. Due to the reduced number of gradient units, patterns 8–12 displayed diminished absorption but still achieved −5 dB within the same frequency range (Fig. 5d). Additionally, to verify the stability of the coded array, reflectivity curves for different letter patterns demonstrated full-band coverage (< -−5 dB) from 2–18 GHz (Fig. S46). For the VIS/IR coding system, an IR camera was employed to decode and switch between two modes, where "0" signifies concealment and "1" denotes display (Fig. 5e, f). The experimental setup enables independent excitation and detection of the visible “NJU” pattern and the infrared “LIR” pattern based on distinct physical mechanisms; under VIS conditions, the top-layer photochromic ink revealed the pattern "NJU," while the hidden "LIR" pattern was thermally generated via an infrared heat source assisted by MXene and PI components and detected via IR imaging (Fig. S47). Thus, through the integration of radar, IR, and VIS coding within a single unit, multispectral compatible camouflage is effortlessly achieved while simultaneously enabling multimodal conversion.

### Multifunctional Application

**Fig. 6:**
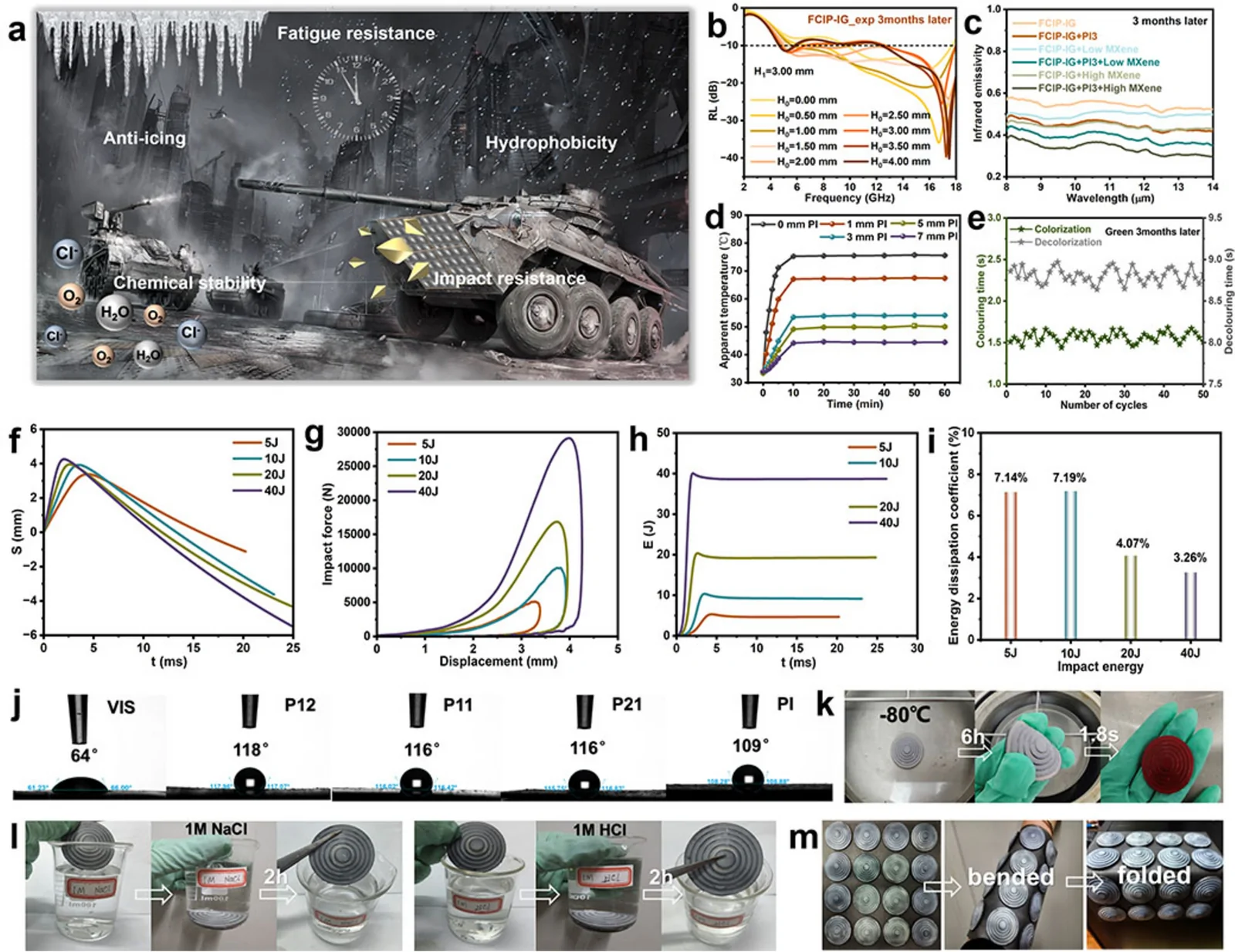
Multifunctional application. **a** Multifunctional scenario application diagram. **b** *RL-f* curve, **c** IR emissivity, **d** Apparent temperature curve, and **e** Coloring and decoloring times of the green photochromic ink within 50 cycles of the device after three months. **f** Displacement–time curve. **g** Impact force–displacement curve. **h** Energy–time curve. **i** Energy dissipation coefficient. **j** Schematic diagram of contact angle. **k** Anti-icing properties of devices at low temperatures. **l** Salt resistance (1 M NaCl, 2 h) and acid resistance (1 M HCl, 2 h) test. **m** Flexibility of the device

Faced with the demands of complex environmental applications, the impact resistance, fatigue resistance, and environmental adaptability of multispectral compatible stealth devices critically influence the survivability and operational efficacy of weaponry (Fig. 6a) [52–54]. To verify the reliability of the device, its stealth performance was reassessed after three months. The results demonstrated exceptional broadband absorption, low emissivity, thermal insulation, and rapid color change performance, unaffected by temperature and humidity variations, indicating the potential for long-term deployment (Figs. 6b–e, S48 and Videos S4–S5). The dynamic impact resistance of the compatible stealth device (Weight: 10 g, Thickness: 7 mm) applied to tank surfaces was evaluated using a drop-weight impact tester (Fig. S49 and Table S5). Force–displacement and displacement–time curves under impact energies of 5–40 J revealed the device’s remarkable elasticity and superior damage resistance under high-impact forces (~ 30,000 N) (Fig. 6f, g). Energy–time curves and corresponding energy dissipation coefficients confirmed the device’s outstanding energy absorption properties, dissipating > 96% of the total impact energy (40 J) (Fig. 6h, i). Such exceptional mechanical performance underscores its suitability for mitigating impact effects on weaponry. The superhydrophobic surface enables self-cleaning via the "lotus effect," preserving compatible stealth functionality. While the top-layer photochromic ink exhibits a relatively low water contact angle (< 90°), the FCIP layer demonstrates robust hydrophobicity (> 115°). Consequently, the overall structure resists corrosion, and the detachable nature of the surface photochromic ink facilitates swift adaptation to diverse environments (Fig. 6j). Following exposure to extreme subzero conditions (−80 °C) for 6 h, the surface remained ice-free, with both visible-light color change and infrared emissivity remaining unchanged, validating the weather resistance of the device (Figs. 6k and S50). After immersion in a 1 M NaCl and HCl solution for 2 h, no significant signs of corrosion were observed on the surface of the device, and performance tests showed favorable results, demonstrating its chemical stability (Figs. 6l and S51). Meanwhile, the radar stealth performance of the IG device also remains stable under these extreme environments (Fig. S52). The integrated components can be freely bent or folded, demonstrating exceptional flexibility (Fig. 6m). Static three-point bending and 1000-cycle fatigue tests further verify its high load-bearing capacity and outstanding bending durability, confirming the mechanical reliability for applications on irregular surfaces (Fig. S53). Thus, the designed radar-IR-VIS compatible stealth device achieves exceptional "environmental immunity," ensuring stable operation under any complex conditions. The designed IG camouflage system exhibits superior performance compared to conventional material systems, featuring broadband absorption, low IR emissivity, thermal insulation, rapid color change, incident-angle insensitivity, programmable and multimodal design, and facile, pollution-free fabrication (Table S6).

## Conclusion

In summary, this work developed a programmable and multifunctional IG metadevice for radar-IR-VIS compatible camouflage. Guided by neural network (NN) predictions for rapid optimization, and validated through electromagnetic (EM) simulations and experiments, the study achieved seamless integration of material assembly, structural design, and device fabrication. The camouflage mechanism relies on the synergistic interaction of IG units, thermal insulation foam, and photochromic inks, achieving MW full-band coverage (2–18 GHz), broad IR temperature modulation (ΔT ≈ 65 °C), low emissivity (0.38), and rapid VIS color change (1 ~ 2 s). Furthermore, the highly rotationally symmetric structure ensures insensitivity to incident angles, enabling omnidirectional response. Independently configurable units allow programmable and multimodal design for switchable information display across VIS and IR. The device’s exceptional impact resistance (~ 30,000 N), chemical stability, fatigue resistance, and weather durability establish a foundation for long-term deployment in harsh environments. By integrating materials science, physics, electronics, and artificial intelligence, this efficient, user-friendly, and environmentally benign design strategy offers a novel pathway to enhance the survivability of equipment across diverse operational scenarios.

## Supplementary Information

Below is the link to the electronic supplementary material.

Supplementary file1 (DOCX 9021 kb)

Supplementary file2 (MP4 1316 kb)

Supplementary file3 (MP4 961 kb)

Supplementary file4 (MP4 359 kb)

Supplementary file5 (MP4 1268 kb)

Supplementary file6 (MP4 417 kb)
