# Supplementary material for: Ultra-Broadband Microwave Absorption and Programmable Multispectral Camouflage Enabled by Neural-Network-Driven Impedance-Gradient Metadevices
Source: Nanomicro Lett. 2026 Jun 16;18:403. doi: 10.1007/s40820-026-02247-z (PMC13272727; doi:10.1007/s40820-026-02247-z)
Supplement: Supplementary file 1 — Supplementary file1 (DOCX 9021 kb) [file 40820_2026_2247_MOESM1_ESM.docx]

Supporting Information for

**Ultra-Broadband Microwave Absorption and Programmable Multispectral Camouflage Enabled by Neural-Network-Driven Impedance-Gradient Metadevices**

Chen Li ^1^, Leilei Liang^3^, Baoshan Zhang ^1 *^, Yi Yang ^1 *^, Guangbin Ji ^2 *^

^1^ School of Electronic Science and Engineering, Nanjing University, Nanjing 210093, P. R. China

^2^ College of Materials Science and Technology, Nanjing University of Aeronautics and Astronautics, Nanjing 210016, P. R. China

^3^ Department of Chemistry, Tsinghua University, Beijing 100084, P. R. China

*Corresponding authors. E-mail: [bszhang@nju.edu.cn](mailto:bszhang@nju.edu.cn) (Baoshan Zhang); [malab@nju.edu.cn](mailto:malab@nju.edu.cn) (Yi Yang); [gbji@nuaa.edu.cn](mailto:gbji@nuaa.edu.cn) (Guangbin Ji)

**Note S1 Preparation process of IG devices and material characterization**

*Materials:* The photochromic ink used in this study was purchased from Shenzhen Huancai Photochromic Technology Co., Ltd. The main functional photochromic components include 3,3-diphenyl-3H-naphtho[2,1-b]pyran (C₂₅H₁₈O) and spiro (2H-indole-2,3'-(3H) naphtha (2,1-B) (1,4) oxazine) derivative (C₃₀H₂₇N₃O), which are responsible for the UV-triggered reversible color change. PI thermal insulation foam was purchased from Jiangsu Environmental Protection New Materials Co., Ltd., FCIP powder was purchased from Jilin Zhuochuang New Materials Co., Ltd., and PDMS and MXene solution were purchased from Nanjing Chemical Reagent Co., Ltd.

FCIP/PDMS mixtures in varying ratios (1:2, 1:1, 2:1) are poured into the molds, set into shape, and demolded after drying to yield the IG structural units. The uppermost two layers of the gradient structure consist of FCIP/PDMS-12, the middle two layers are FCIP/PDMS-11, and the bottom two layers are FCIP/PDMS-21. Subsequently, an additional layer of FCIP/PDMS-21 film and PI foam is introduced at the base of each unit, while a modulated MXene-doped photochromic ink is coated atop the unit.

Corresponding SEM cross-sectional and planar images vividly illustrate the impedance gradation, with the proportion of FCIP particles within the PDMS progressively increasing. The PI foam displays a stratified, porous morphology with a smooth surface (Figs. S2 and S3). Further analysis of FCIP reveals its flake-like structure (d = 6.49 μm) and a loose density of 1.24 g/cm³ (Figs. S4 and S5). The EDS and elemental mapping provide comprehensive distribution patterns and quantitative ratios, while the characteristic Fe (06-0696) peaks in XRD patterns, coupled with distinct -OH peaks in FTIR spectra, conclusively verify the exclusive presence of FCIP (Fig. S6). XPS provides a deeper insight into the chemical states of FCIP, including Fe 2p, O 1s, and C 1s (Fig. S7a-d). Magnetic property characterization reveals a saturation magnetization (*M_s_*) as high as 230.2 emu/g, which is conducive to enhanced magnetic loss capability (Fig. S7e). Additionally, the UV-VIS-NIR spectrum displays the absorptivity and reflectivity of FCIP (Fig. S7f).

**Note S2 Microwave absorption performance**

For the flexible P structure, FCIP/PDMS-21 achieves an EAB of 9.16 GHz at a thickness of 1.49 mm (Fig. S8a-c). Notably, as the FCIP content increases, the absorption curves progressively shift toward lower frequencies, with FCIP/PDMS-21 exhibiting the *RL_min_* of -55.87 dB at 6.00 GHz (Fig. S8d-f). Simultaneously, the complex permittivity ($\varepsilon$) and permeability ($\mu$) curves exhibit a regular ascending trend, with magnetic loss predominating, including natural resonance and eddy current losses (Fig. S9a-f). Dielectric loss arises primarily from polarization loss rather than conductive loss (Figs. S9g-i and S10), as detailed in the supporting theoretical formulations (Note S3). An analysis of the impedance matching for FCIP/PDMS-12, FCIP/PDMS-11, and FCIP/PDMS-21 reveals gradual trends (Fig. S11a-c). The corresponding attenuation coefficient ($\alpha$) curves strongly validate the superior loss capability of FCIP/PDMS-21, with $\alpha$ reaching a maximum of 420 (Fig. S11d).

**Note S3 Theoretical analyses of relevant parameters**

The thickness (*d*) of the absorber layer can be expressed as:

$\text{d}=\frac{\lambda_{0}}{4\sqrt{\mu_{r}\varepsilon_{r}}}$ (S-1)

The complex permeability ($\mu$) and complex permittivity ($\varepsilon$) can be expressed as:

$\mu_{r}=\mu'-j\mu''$ (S-2)

$\varepsilon_{r}=\varepsilon'-j\varepsilon''$ (S-3)

Impedance matching (*Z*) of the multi-layer structure can be expressed as:

(S-4)

(S-5)

$Z=\left|\frac{Z_{in}^{\left(1\right)}}{Z_{0}}\right|$ (S-6)

where $\gamma_{i}=\alpha_{i}+j\beta_{i}=j\frac{2\pi}{\lambda_{0}}\sqrt{\mu_{r,i}\varepsilon_{r,i}}$ is the propagation constant, metal backplane$Z_{in}^{\left(N\right)}=0$, surface layer input impedance is $Z_{in}^{\left(1\right)}=0$.

The reflection loss (*RL*) can be expressed by the following equation:

$RL=\frac{Z_{in}^{\left(1\right)}-Z_{0}}{Z_{in}^{\left(1\right)}+Z_{0}}$ (S-7)

where *Z_in_* and *Z_0_* are impedance of input impedance and free space of the absorber.

Attenuation constant ($\alpha$) can be expressed as:

$\alpha_{i}=\frac{\sqrt{2}\pi f}{c}\sqrt{\left(\mu''_{i}\varepsilon''_{i}-\mu'_{i}\varepsilon'_{i}\right)+\sqrt{\left(\mu''_{i}\varepsilon''_{i}-\mu'_{i}\varepsilon'_{i}\right)^{2}+\left(\varepsilon'_{i}\mu''_{i}+\varepsilon''_{i}\mu'_{i}\right)^{2}}}$ (S-8)

The dielectric loss tangent ($\tan\delta_{\varepsilon}$) and magnetic loss tangent ($\tan\delta_{\mu}$) can be expressed as:

$\tan\delta_{\varepsilon}=\frac{\varepsilon"}{\varepsilon'}$ (S-9)

$\tan\delta_{\mu}=\frac{\mu"}{\mu'}$ (S-10)

Polarization effect can be evaluated using the Debye equation:

$\left(\varepsilon'-\frac{\varepsilon_{s}+\varepsilon_{\infty}}{2}\right)^{2}+\left(\varepsilon''\right)^{2}=\left(\frac{\varepsilon_{s}-\varepsilon_{\infty}}{2}\right)^{2}$ (S-11)

$\varepsilon''=\varepsilon_{p}''+\varepsilon_{c}''=\varepsilon_{p}''+\frac{\sigma}{2\pi f\varepsilon_{0}}$ (S-12)

where $\varepsilon_{\infty}$ and $\varepsilon_{s}$ are the relative dielectric permittivity at the high frequency limit and static permittivity. $\varepsilon_{p}$ and $\varepsilon_{c}$ are polarization loss and conductivity loss, $\sigma$ is conductivity. The resulting semicircle from the equation is known as a Cole-Cole semicircle, representing a Debye relaxation process.

The root mean square error (RMSE) of the convolutional neural network (NN) fitting model can be expressed as:

$RMSE=\sqrt{\frac{1}{N}\sum_{i=1}^{N} \left(y_{i}-\mathop{y_{i}}\limits^{\wedge} \right)^{2}}$ (S-13)

where *N* is the number of predicted values, $y_{i}$ and $\mathop{y_{i}}\limits^{\wedge}$are the true value and predicted value of the integral value of *RL* (-10 dB) within the range of 2-18 GHz.

IR radiant energy adheres to the Stefan-Boltzmann law:

$W=\varepsilon\sigma T^{4}$ (S-14)

The total radiative energy of an object is denoted as *W*, where $\sigma$ represents the Stefan-Boltzmann constant, $\varepsilon$ signifies the IR emissivity of object, and *T* stands for the absolute temperature of the object.

Radar cross-section (RCS) can be expressed as:

$\sigma\left(dB\cdot m^{2}\right)=10\lg\left(4\pi\lim_{R\to\infty} R^{2}\frac{\left|E^{s}\right|^{2}}{\left|E^{i}\right|^{2}}\right)$ (S-15)

where $E^{s}$ and $E^{i}$ represent the intensities of the scattered electric field and incident electric field, respectively, and *R* is the detection distance.

**Note S4 Electromagnetic simulation (CST)**

Full-wave electromagnetic simulations were performed in CST Studio Suite to optimize the structural parameters of impedance-gradient metadevices for broadband absorption. The frequency-domain solver (Finite Element Method, FEM) was employed with multiple electric field monitors. A frequency sweep from 2 to 18 GHz was configured with periodic boundaries in the x-y plane. The structure incorporated a metallic ground plane at the bottom layer, while plane wave excitation propagated along the -z axis toward the device surface.

**Note S5 Optical simulation (FDTD)**

Optical simulations were performed to analyze infrared reflectance and transmittance across the 8-14 μm spectral range while simultaneously monitoring electric field distributions. The Finite-Difference Time-Domain (FDTD) method was implemented with plane wave excitation and field monitors. Structural parameters of CIP were derived from experimental SEM characterization, while optical properties were assigned based on Fe data from the RefractiveIndex. INFO database.

**Note S6 NRL Arch measurement**

The NRL Arch measurement setup includes a transmitting antenna and a receiving antenna, both fixed on a large arched structure. The sample to be tested is placed on a rotating platform below the center of the arch. By moving the antennas along the arch and rotating the sample, the reflectivity of the material can be quickly measured at different incidence angles (0-90°) and polarization angles (0-180°). The sample size is 180 mm🞨180 mm. Calibration is performed using a metal plate: a flat, smooth metal plate (a perfect electrical conductor) of the same size as the sample is placed at the sample location, and its reflected signal is measured as the response of an ideal perfect reflector.

**Note S7 Complete neural network training process**

The complete neural network training process is as follows:

**1. Define 15 sets of data**

new_data = [

1, 1, 1, 157.38;

1, 1, 2, 175.94;

1, 1, 3, 211.68;

1, 1, 4, 259.72;

1, 2, 1, 176.83;

1, 3, 1, 201.98;

1, 4, 1, 231.69;

2, 1, 1, 161.69;

2, 2, 1, 178.82;

2, 2, 2, 212.01;

2, 2, 3, 269.78;

2, 2, 4, 299.35;

2, 2, 5, 298.65;

2, 2, 6, 276.68;

3, 1, 1, 164.51;];

X = new_data(:, 1:3);

Y = new_data(:, 4);

**2. Data normalization - Detailed record of the normalization process**

fprintf('============ Explanation of the data normalization process ============\n');

fprintf('Original data range：\n');

fprintf('h1: [%.0f, %.0f]\n', min(X(:,1)), max(X(:,1)));

fprintf('h2: [%.0f, %.0f]\n', min(X(:,2)), max(X(:,2)));

fprintf('h3: [%.0f, %.0f]\n', min(X(:,3)), max(X(:,3)));

fprintf('RL: [%.2f, %.2f] dB\n', min(Y), max(Y));

fprintf('RL mean: %.2f dB\n', mean(Y));

% Normalisation of X (input features) - mapped to the interval [0,1]

[X_norm, X_norm_settings] = mapminmax(X', 0, 1);

% Normalisation for Y (output labels) – Divide the RL value by 100 to unify the scale.

scale_factor = 100; % Scaling factor: Converting RL from the hundreds place to the units place range

[Y_norm, Y_norm_settings] = mapminmax(Y', 0, 1);

% Explicitly display the normalisation method by dividing by 100 (for ease of understanding)

Y_norm_custom = Y' / scale_factor;

fprintf('\n Normalisation strategy：\n');

fprintf('X Normalisation：Maximum-minimum normalisation to the interval [0,1] \n');

fprintf('Y Normalisation：RL value divided by scaling factor %.0f (uniform dimensions)\n', scale_factor);

fprintf('Normalised RL range：[%.4f, %.4f]\n', min(Y_norm_custom), max(Y_norm_custom));

fprintf('Normalised mean RL：%.4f\n', mean(Y_norm_custom));

fprintf('============================================\n\n');

**3. Creating a neural network**

net = fitnet([10, 10], 'trainlm');

net.layers{1}.transferFcn = 'tansig';

net.layers{2}.transferFcn = 'tansig';

net.layers{3}.transferFcn = 'purelin';

net.trainParam.epochs = 1000;

net.trainParam.lr = 0.01;

**4. Training neural networks**

[net, tr] = train(net, X_norm, Y_norm);

**5. Calculate the predicted values for the training set and renormalise them**

Y_pred_norm = net(X_norm); % Prediction (normalised value)

Y_pred = mapminmax('reverse', Y_pred_norm, Y_norm_settings); % De-normalisation

**6. Detailed Explanation of the RMSE Calculation Process**

% Calculate the Root Mean Square Error on a normalised scale

rmse_norm = sqrt(mean((Y_norm - Y_pred_norm).^2));

% Calculate the root mean square error on the original scale

rmse_original = sqrt(mean((Y - Y_pred').^2));

fprintf('============ Explanation of the RMSE Calculation Process ============\n');

fprintf(' Note: Neural networks are trained and make predictions on normalised data\n');

fprintf('\n1. Error on the normalised scale：\n');

fprintf('Normalised Root Mean Square Error = %.6f (based on the [0,1] interval)\n', rmse_norm);

fprintf('This value reflects the fitting accuracy of the normalised data \n');

fprintf('\n2. Re-normalize to the original scale：\n');

fprintf('Original scale RMSE = %.4f dB\n', rmse_original);

fprintf('Equivalent calculation：%.6f × %.0f ≈ %.4f dB\n', rmse_norm, scale_factor, rmse_norm*scale_factor);

fprintf('\n3. Error Analysis (Original Scale)：\n');

fprintf('RL mean = %.2f dB\n', mean(Y));

fprintf('Absolute error = %.2f dB\n', rmse_original);

fprintf('Relative error = %.2f%% (RMSE/mean)\n', (rmse_original/mean(Y))*100);

% Verify the consistency of normalisation

if abs(rmse_original - rmse_norm*scale_factor) < 1.0

fprintf('\n✓ Verification passed: Normalised RMSE × %.0f ≈ Original-scale RMSE \n', scale_factor);

fprintf('Sources of error: Minor discrepancies between mapminmax and linear scaling \n');

else

fprintf('\n⚠ Note: Differences exist in normalisation methods \n');

end

fprintf('===========================================\n\n');

**7. Plot a comparison chart of actual values versus forecast values**

figure;

plot(Y, 'bo-', 'LineWidth', 1.5, 'MarkerSize', 8, 'DisplayName', 'actual RL values');

hold on;

plot(Y_pred, 'rs--', 'LineWidth', 1.5, 'MarkerSize', 8, 'DisplayName', 'Predict RL values ');

hold off;

xlabel('Sample Index');

ylabel('RL (dB)');

title('Comparison of Neural Network Prediction Results (Original Scale)');

legend('show', 'Location', 'best');

grid on;

**8. Plotting the training loss curve**

figure;

plot(tr.perf);

xlabel('Training cycle (Epochs)');

ylabel('mean square error (MSE)');

title('Neural Network Training Convergence Curve (Normalised Scale)');

grid on;

**9. Predicting RL values for specified parameters (h3=2.93, h2=3.84, h1=5.00)**

h_specified = [2.93, 3.84, 5.00];

% Normalise the specified parameters

h_norm = mapminmax('apply', h_specified', X_norm_settings);

% Using neural networks for prediction

predicted_RL_norm = net(h_norm);

% De-normalisation yields the actual RL value

predicted_RL_value = mapminmax('reverse', predicted_RL_norm, Y_norm_settings);

fprintf('============ Parameter-specific prediction ============\n');

fprintf('Input parameters：\n');

fprintf('h1 = %.2f, h2 = %.2f, h3 = %.2f\n', h_specified);

fprintf('Prediction results：\n');

fprintf('Normalised predicted values：%.4f\n', predicted_RL_norm);

fprintf('Original scale predicted value：%.2f dB\n', predicted_RL_value);

fprintf('=====================================\n\n');

**10. Genetic algorithm optimisation for finding optimal parameters**

fprintf('Commencing genetic algorithm optimisation...\n');

objFunc = @(h) -mapminmax('reverse', net(mapminmax('apply', h', X_norm_settings)), Y_norm_settings);

lb = [0, 0, 0];

ub = [5, 5, 5];

options = optimoptions('ga', 'PopulationSize', 100, 'MaxGenerations', 200, 'Display', 'iter');

best_h = ga(objFunc, 3, [], [], [], [], lb, ub, [], options);

**11. Output optimisation results**

predicted_RL_opt = -objFunc(best_h);

fprintf('\n============ Genetic algorithm optimisation results ============\n');

fprintf('Optimal parameter combination：\n');

fprintf('h1 = %.4f, h2 = %.4f, h3 = %.4f\n', best_h(1), best_h(2), best_h(3));

fprintf('Predicted maximum reflection loss：\n');

fprintf('RL = %.2f dB\n', predicted_RL_opt);

fprintf('=========================================\n');

% Plot the optimisation results

figure;

subplot(1,2,1);

bar(best_h);

set(gca, 'XTickLabel', {'h1', 'h2', 'h3'});

ylabel('Parameter value ');

title('Parameters optimised by genetic algorithms');

grid on;

subplot(1,2,2);

bar(predicted_RL_opt);

ylabel('RL (dB)');

title('Predicted maximum RL');

ylim([0, 350]);

grid on;

**12. Present all forecast results in summary form**

figure;

plot(Y, 'bo-', 'LineWidth', 1.5, 'MarkerSize', 8, 'DisplayName', 'Actual RL value');

hold on;

plot(Y_pred, 'rs--', 'LineWidth', 1.5, 'MarkerSize', 8, 'DisplayName', 'Neural network prediction');

plot(16, predicted_RL_value, 'g^', 'MarkerSize', 12, 'LineWidth', 2, ...

'DisplayName', sprintf('Parameter-specific prediction: %.2f dB', predicted_RL_value));

plot(17, predicted_RL_opt, 'md', 'MarkerSize', 12, 'LineWidth', 2, ...

'DisplayName', sprintf('Optimised Parameter Prediction: %.2f dB', predicted_RL_opt));

hold off;

xlabel('Sample Index');

ylabel('RL (dB)');

title('Complete Summary of Forecast Results');

legend('show', 'Location', 'best');

grid on;

**13. End of programme prompt**

fprintf('\n================ Program execution completed ================\n');

fprintf('Summary of Key Results：\n');

fprintf('1. Neural network training completed, RMSE = 0.2f dB (relative error 0.2f%)\n', rmse_original, (rmse_original/mean(Y))*100);

fprintf('2. Parameter-specific prediction：h=(%.2f,%.2f,%.2f) -> RL=%.2f dB\n', h_specified, predicted_RL_value);

fprintf('3. Optimised Parameter Prediction：h=(%.4f,%.4f,%.4f) -> RL=%.2f dB\n', best_h, predicted_RL_opt);

fprintf('============================================\n');

**Table S1** Predicted values and actual values

| **Predicted** | 159.065 | 177.850 | 213.611 | 261.741 | 177.419 | 202.185 | 214.349 | 162.402 | 191.062 | 226.520 | 271.780 | 296.792 | 301.952 | 279.698 | 163.870 |
| --- | --- | --- | --- | --- | --- | --- | --- | --- | --- | --- | --- | --- | --- | --- | --- |
| **Actual** | 157.380 | 175.940 | 211.680 | 259.720 | 176.830 | 201.980 | 231.690 | 161.690 | 178.820 | 212.010 | 269.780 | 299.350 | 298.650 | 276.680 | 164.510 |

**Table S2** Comparison of IR emissivity between the material has been reported and our work

| **Names of Sample** | **Emissivity** | **Ref.** |
| --- | --- | --- |
| PVDF/Ti_3_C_2_T_x_/h-CFO | 0.51 | [S1] |
| C-BN/Glass | 0.42 | [S2] |
| AZO NCs | 0.61 | [S3] |
| TDIE regulators | 0.41 | [S4] |
| CS@MoSe_2_ | 0.32 | [S5] |
| ITO/SiO_2_/ITO | 0.42 | [S6] |
| ITO/HfO_2_/ITO | 0.38 | [S7] |
| LAMs | 0.24 | [S8] |
| $\pi$-conjugated MOFs | 0.21 | [S9] |
| AlPO_4_/NiFe@CNT | 0.80 | [S10] |
| Photonic Battery | 0.53 | [S11] |
| MCSA | 0.16 | [S12] |
| Thermomechanical expressway | 0.11 | [S13] |
| Stealth metacoating | 0.46 | [S14] |
| AgNW@C | 0.28 | [S15] |
| NC/Ni(HS) | 0.78 | [S16] |
| Porous carbon aerogel | 0.52 | [S17] |
| MDP | 0.34 | [S18] |
| ATO-Ag-BFs | 0.68 | [S19] |
| TiB_2_-Al_2_O_3_-TiB_2_ | 0.44 | [S20] |
| IG | 0.63 | This work |
| IG+PI-3 | 0.49 | This work |
| IG+Low MXene | 0.54 | This work |
| IG+High MXene | 0.47 | This work |
| IG+PI-3+Low MXene | 0.43 | This work |
| IG+PI-3+High MXene | 0.38 | This work |

**Table S3** The sensitivity parameter value when capturing camouflage image

|  | 8:00 | 9:00 | 10:00 | 11:00 | 12:00 | 13:00 | 14:00 | 15:00 | 16:00 | 17:00 |
| --- | --- | --- | --- | --- | --- | --- | --- | --- | --- | --- |
| Yellow  (weak) | 50 | 50 | 50 | 50 | 50 | 72 | 72 | 72 | 67 | 61 |
| Yellow  (strong) | 72 | 72 | 72 | 72 | 72 | 72 | 72 | 72 | 67 | 61 |
| Green  (weak) | 50 | 50 | 50 | 50 | 50 | 50 | 50 | 50 | 68 | 61 |
| Green  (strong) | 72 | 72 | 72 | 72 | 72 | 72 | 71 | 72 | 67 | 61 |

**Table S4** Reduction in RCS_max_ under different coding sequences

| $\boldsymbol{\Delta}$**RCS_max_ (dB·m²)** | **2 GHz** | **5 GHz** | **10 GHz** | **15 GHz** | **18 GHz** |
| --- | --- | --- | --- | --- | --- |
| Pattern 1 | 2.86 | 11.38 | 15.81 | 26.45 | 33.09 |
| Pattern 2 | 2.78 | 11.43 | 15.77 | 26.42 | 33.00 |
| Pattern 3 | 2.78 | 11.43 | 15.77 | 26.41 | 33.01 |
| Pattern 4 | 2.76 | 11.38 | 15.81 | 26.43 | 33.07 |
| Pattern 5 | 2.38 | 9.01 | 13.58 | 22.81 | 20.78 |
| Pattern 6 | 2.49 | 8.92 | 13.57 | 22.69 | 20.76 |
| Pattern 7 | 2.39 | 8.99 | 13.53 | 22.60 | 20.71 |
| Pattern 8 | 2.63 | 8.90 | 13.66 | 22.78 | 20.83 |
| Pattern 9 | 1.79 | 6.28 | 9.87 | 14.89 | 14.40 |
| Pattern 10 | 1.91 | 6.15 | 9.95 | 14.85 | 14.41 |
| Pattern 11 | 1.97 | 6.21 | 9.96 | 14.82 | 14.44 |
| Pattern 12 | 1.84 | 6.23 | 9.88 | 14.86 | 14.42 |

**Table S5** Parameters related to the drop hammer test

| **E_Sample_ (J)** | **M_total_ (kg)** | **V_impact_ (m/s)** | **E_impact_ (J)** | **H_fall_ (mm)** | **V_0_ (m/s)** | **E_measure_ (J)** | **F_peak_ (N)** | **E_peak_ (J)** | **S_end_ (mm)** | **E_end_ (J)** | **V_rebound_ (m/s)** | **E_rebound_ (J)** |
| --- | --- | --- | --- | --- | --- | --- | --- | --- | --- | --- | --- | --- |
| 5 J | 9.239 | 1.04 | 5 | 55 | 1.04 | 4.99 | 5102.80 | 4.78 | 2.007 | 4.643 | 0.13 | 0.078 |
| 10 J | 9.239 | 1.47 | 10 | 110 | 1.47 | 9.982 | 10069.14 | 9.30 | 2.138 | 9.281 | 0.40 | 0.739 |
| 20 J | 9.239 | 2.08 | 20 | 221 | 2.08 | 19.99 | 16830.16 | 16.92 | 2.583 | 19.186 | 0.59 | 1.608 |
| 40 J | 9.239 | 2.94 | 40 | 441 | 2.93 | 39.66 | 29118.82 | 32.91 | 2.969 | 38.698 | 0.73 | 2.462 |

**Table S6** Comparison of conventional compatible camouflage material systems and our work

| **Sample** | **EAB (GHz)** | **Angle-insensitive** | $\boldsymbol{\Delta}$**RCS (dB·m^2^)** | **Thermal insulation ($\boldsymbol{\Delta T/℃}$)** | **IR emissivity** | **Visible camouflage** | **Multifunctionality** | **Ref.** |
| --- | --- | --- | --- | --- | --- | --- | --- | --- |
| LAM | **🞪** | **🞪** | **🞪** | 50.00 | 0.24 | **🗸** | **🞪** | [S8] |
| AlPO_4_/NiFe@CNT | 4.70 | **🞪** | **🞪** | 36.80 | 0.80 | **🞪** | Flame retardancy | [S10] |
| AgNW@C aerogel | 8.80 | **🞪** | 21.90 | 150.00 | 0.28 | **🞪** | **🞪** | [S15] |
| NC/Ni(HS) | 6.16 | **🞪** | 31.20 | **🞪** | 0.79 | **🞪** | **🞪** | [S16] |
| Flexible plasmonic film | **🞪** | **🞪** | **🞪** | 236.30 | 0.17 | **🗸** | **🞪** | [S21] |
| PMLC aerogel | 7.00 | **🞪** | **🞪** | 240.00 | 0.78 | **🞪** | Stability | [S22] |
| Photonic structures | **🞪** | **🞪** | **🞪** | 15.00 | 0.33 | **🗸** | Flexible | [S23] |
| GST film | **🞪** | **🞪** | **🞪** | 140.00 | 0.08 | **🗸** | Flexible | [S24] |
| Hierarchical metamaterials | 9.60 | **🞪** | **🞪** | **🞪** | 0.29 | **🞪** | **🞪** | [S25] |
| Plasmonic metasurface | **🞪** | **🞪** | **🞪** | **🞪** | 0.11 | **🞪** | Mechanical flexibility  Robustness  Hydrophobic | [S26] |
| MHM | 17.30 | **🞪** | **🞪** | **🞪** | 0.20 | **🞪** | **🞪** | [S27] |
| Metadevice | 6.10 | **🞪** | **🞪** | Dynamic | **🞪** | **🞪** | **🞪** | [S28] |
| FCCM | 4.00 | **🞪** | **🞪** | **🞪** | 0.05-0.85 | **🗸** | Flexible | [S29] |
| Asymmetric metasurface | 10.00 | **🞪** | 10.00 | 55.00 | 0.28 | **🞪** | **🞪** | [S30] |
| Ge/ZnS metamaterial | **🞪** | **🞪** | **🞪** | **🞪** | 0.20 | **🗸** | **🞪** | [S31] |
| Meta-tape | **🞪** | **🞪** | **🞪** | **🞪** | 0.38 | **🗸** | Flexible | [S32] |
| MRM | **🞪** | **🞪** | **🞪** | **🞪** | 0.51 | **🞪** | Information encryption | [S33] |
| Transparent metamaterial | 13.60 | **🞪** | **🞪** | **🞪** | 0.35 | **🞪** | Robustness | [S34] |
| SiO_2_/C@SiC/SiO_2_ | 4.00 | **🞪** | **🞪** | 156.00 | **🞪** | **🞪** | **🞪** | [S35] |
| MXene-based films | **🞪** | **🞪** | **🞪** | **🞪** | 0.10 | **🗸** | **🞪** | [S36] |
| Co@NCNTs-CM | 5.52 | **🞪** | 6.80 | 63.10 | **🞪** | **🞪** | Flame retardancy | [S37] |
| MSKA | 7.20 | **🞪** | 35.69 | 123.10 | **🞪** | **🞪** | **🞪** | [S38] |
| MXene/TiO_2_ | 5.40 | **🞪** | **🞪** | 11.40 | 0.20 | **🞪** | **🞪** | [S39] |
| ZnS/Ge@Cu-ITO-Cu | 4.00 | **🞪** | **🞪** | 93.20 | 0.12 | **🗸** | **🞪** | [S40] |
| IG devices | 16.00 | **🗸** | 41.17 | 65.00 | 0.38 | **🗸** | Programmable  Information encryption Impact resistance  Chemical stability  Fatigue resistance  Weather durability | This work |

**Supplementary Figures**

**
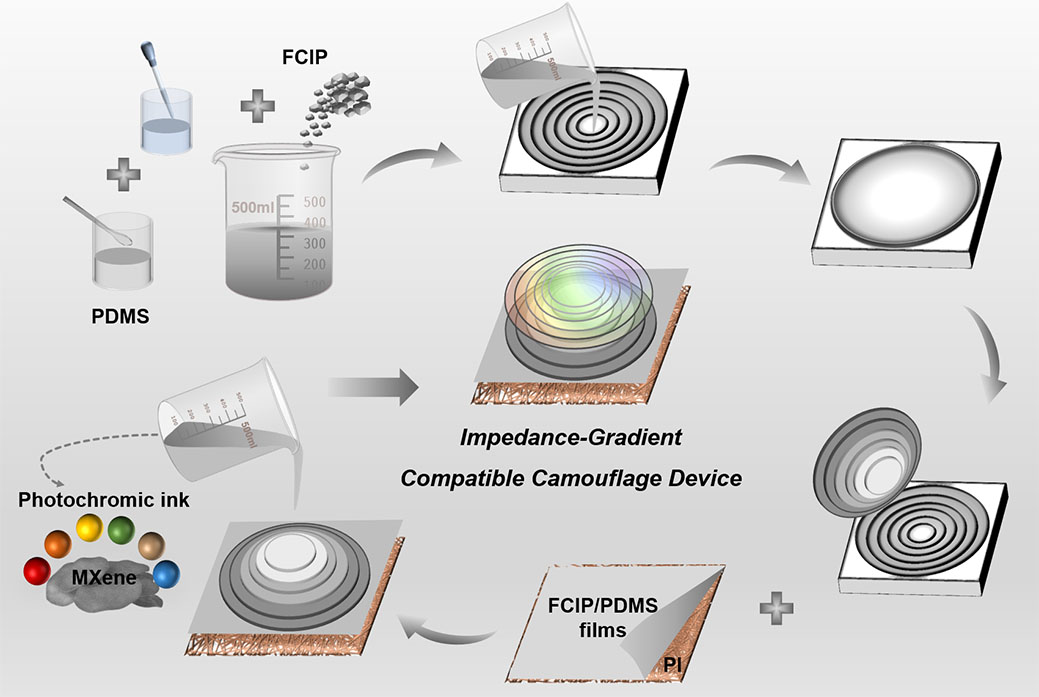
**

**Fig. S1** The design process of the IG metadevice

**
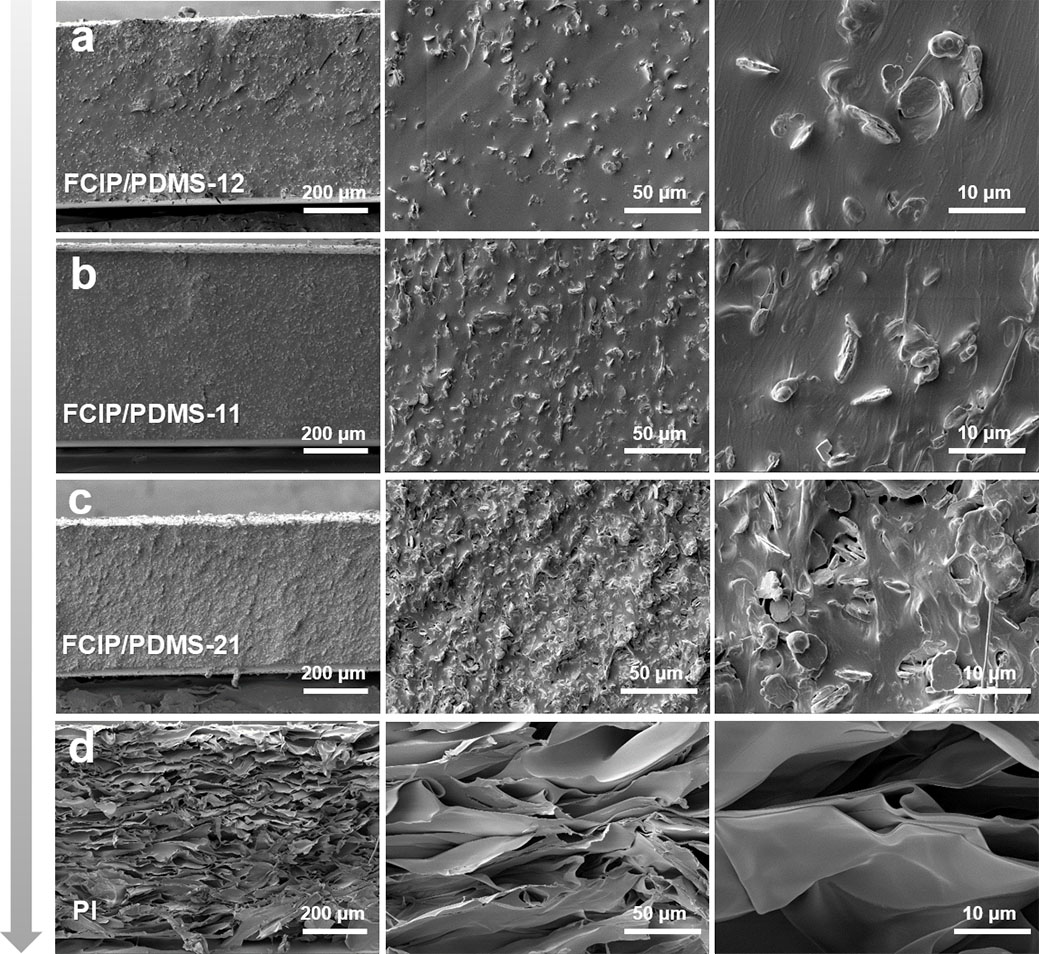
**

**Fig. S2** Typical cross-section SEM images of **a** FCIP/PDMS-12, **b** FCIP/PDMS-12, **c** FCIP/PDMS-12, and d) PI

**
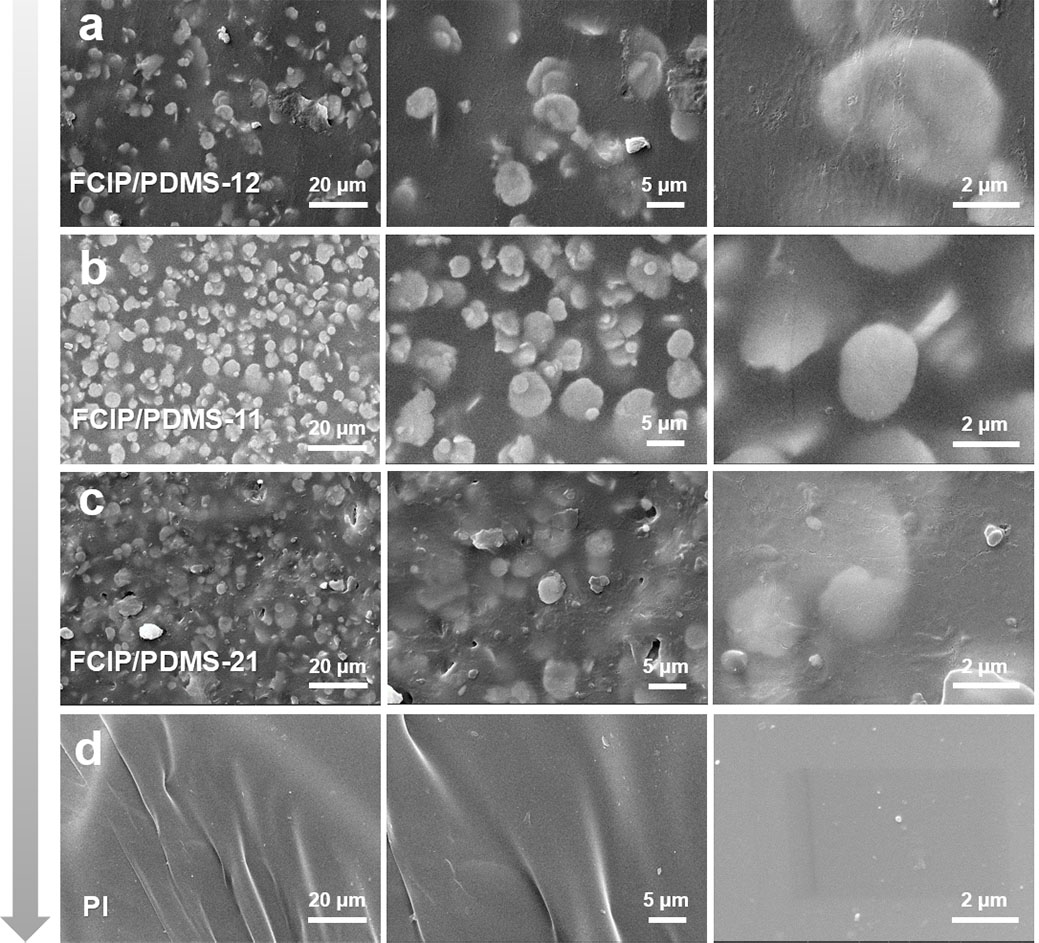
**

**Fig. S3** Typical SEM images of **a** FCIP/PDMS-12, **b** FCIP/PDMS-12, **c** FCIP/PDMS-12, and **d** PI

**
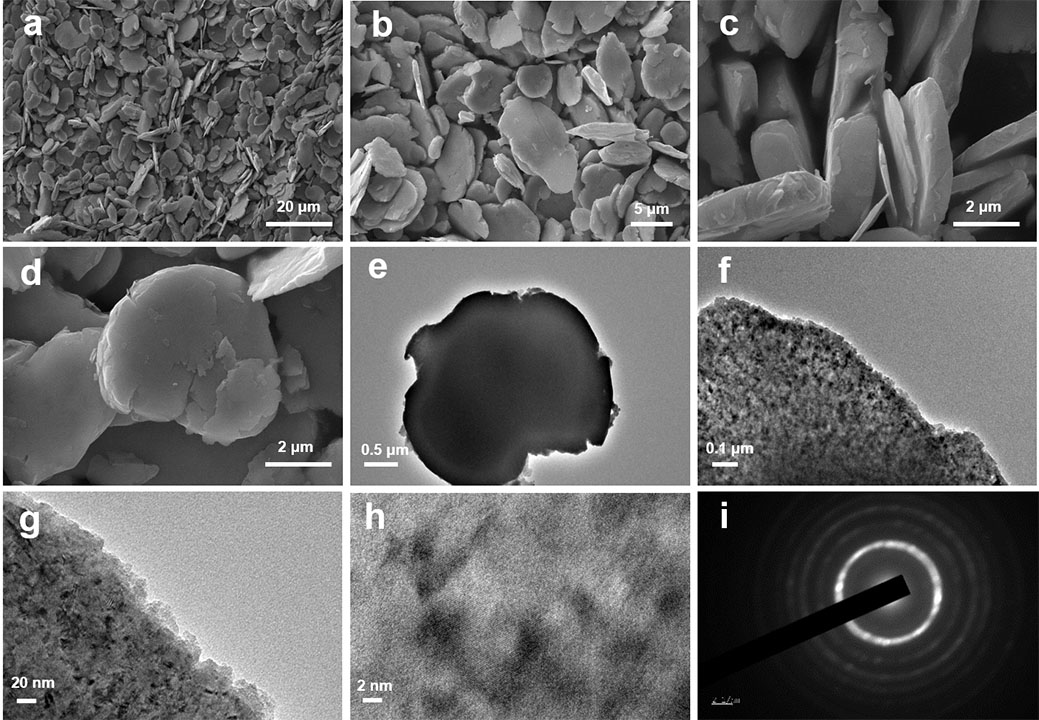
**

**Fig. S4 a-d** Typical SEM images of FCIP. **e-i** Typical TEM images of FCIP

**
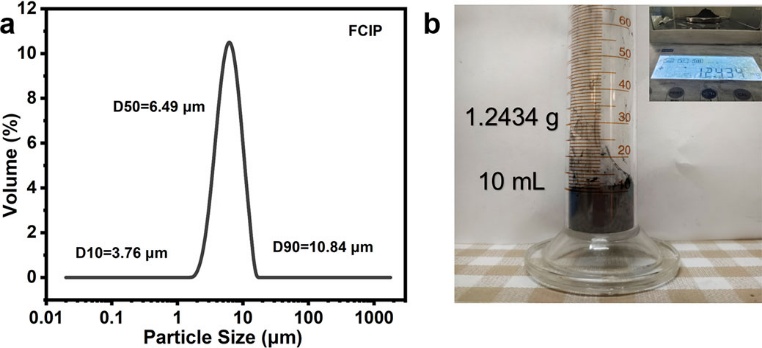
**

**Fig. S5 a** The particle size distribution of FCIP. **b** Schematic diagram of loose density measurement device

**
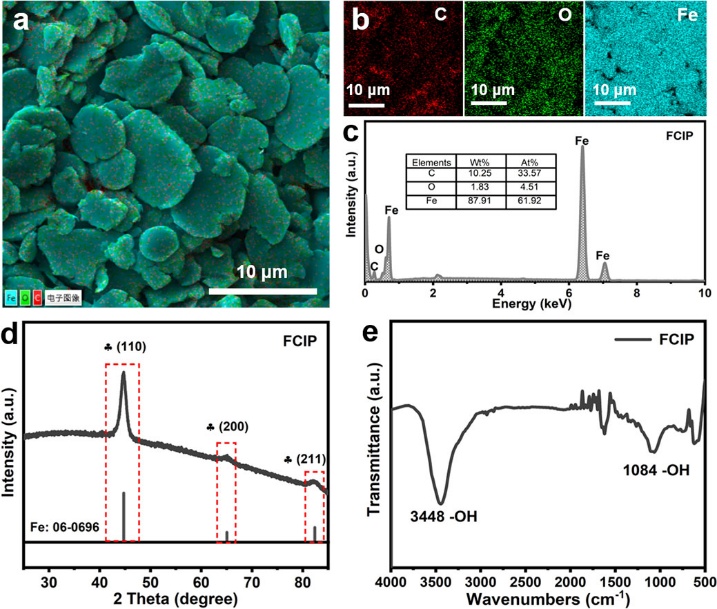
**

**Fig. S6 a** The mapping images of FCIP. **b** The corresponnding elements distribution of C, O, and Fe. **c** EDS spectrum of FCIP **d** XRD pattern of FCIP. **e** FTIR plot of FCIP

**
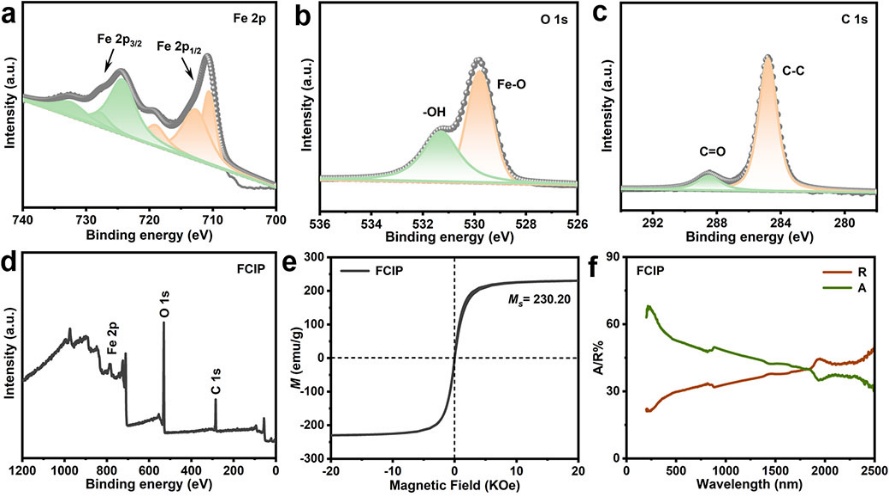
**

**Fig. S7** High resolution spectra for **a** Fe 2p, **b** O 1s, and **c** C 1s. **d** XPS spectrum of FCIP. **e** VSM curves of FCIP. **f** Absorptivity and reflectivity spectra of FCIP

**
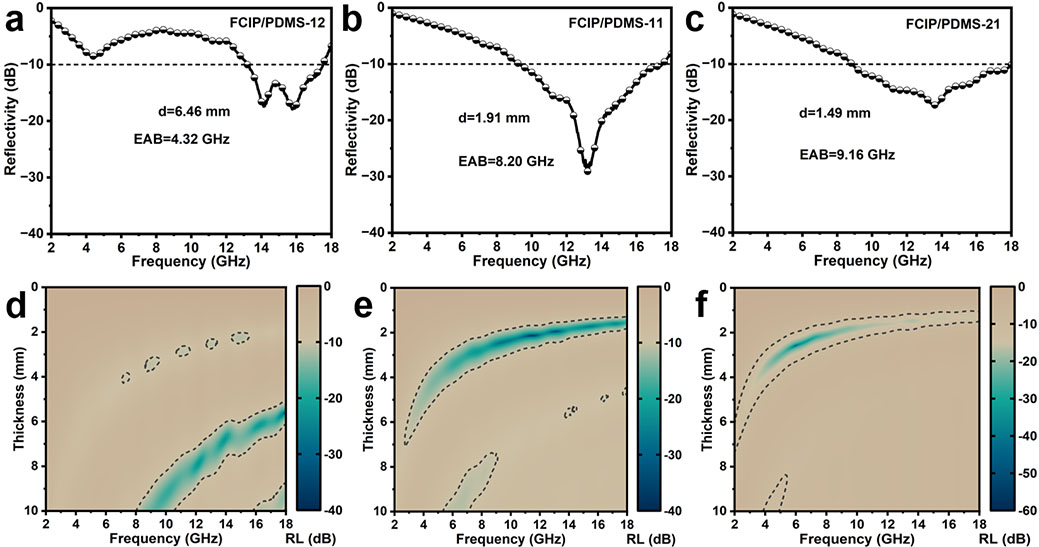
**

**Fig. S8** The typical curves and 2D color maps of **a, d** FCIP/PDMS-12, **b, e** FCIP/PDMS-11, and **c, f** FCIP/PDMS-21

**
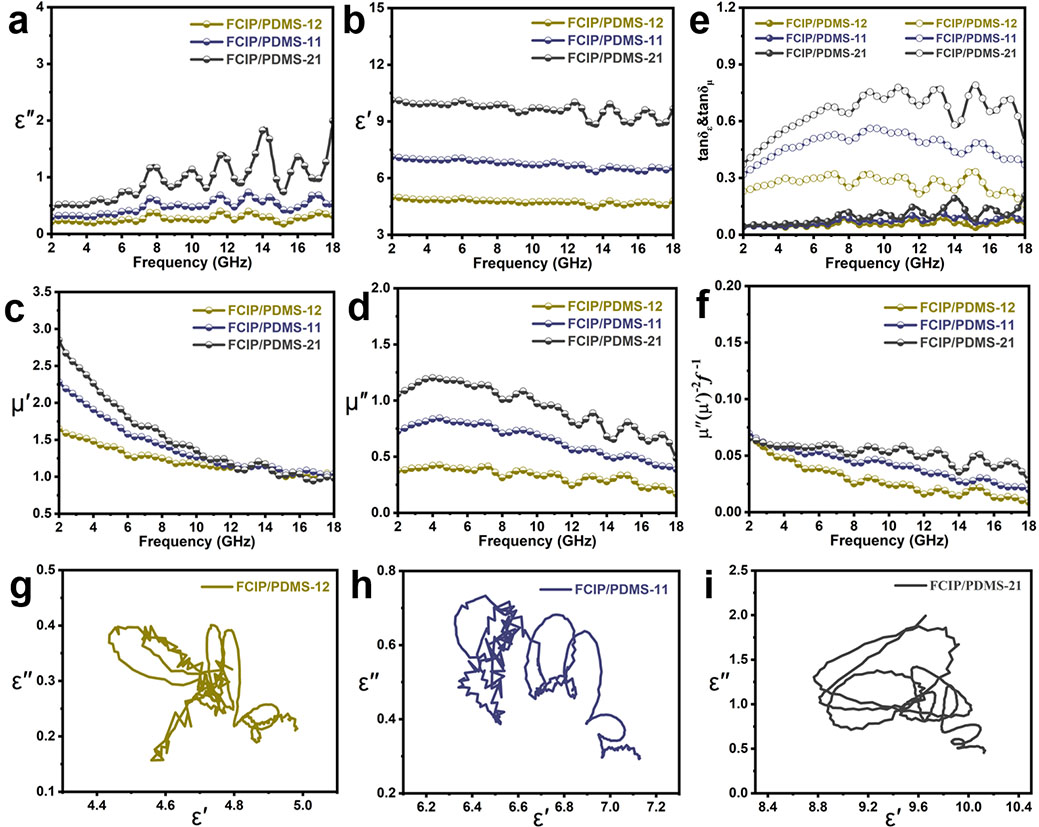
**

**Fig. S9 a, b** Complex permittivity, **c, d** Complex permeability, **e** Dielectric and magnetic loss tangent, **f** C_0_ curves, and **g-i** Cole-Cole curves of FCIP/PDMS-12, FCIP/PDMS-11, and FCIP/PDMS-21

**
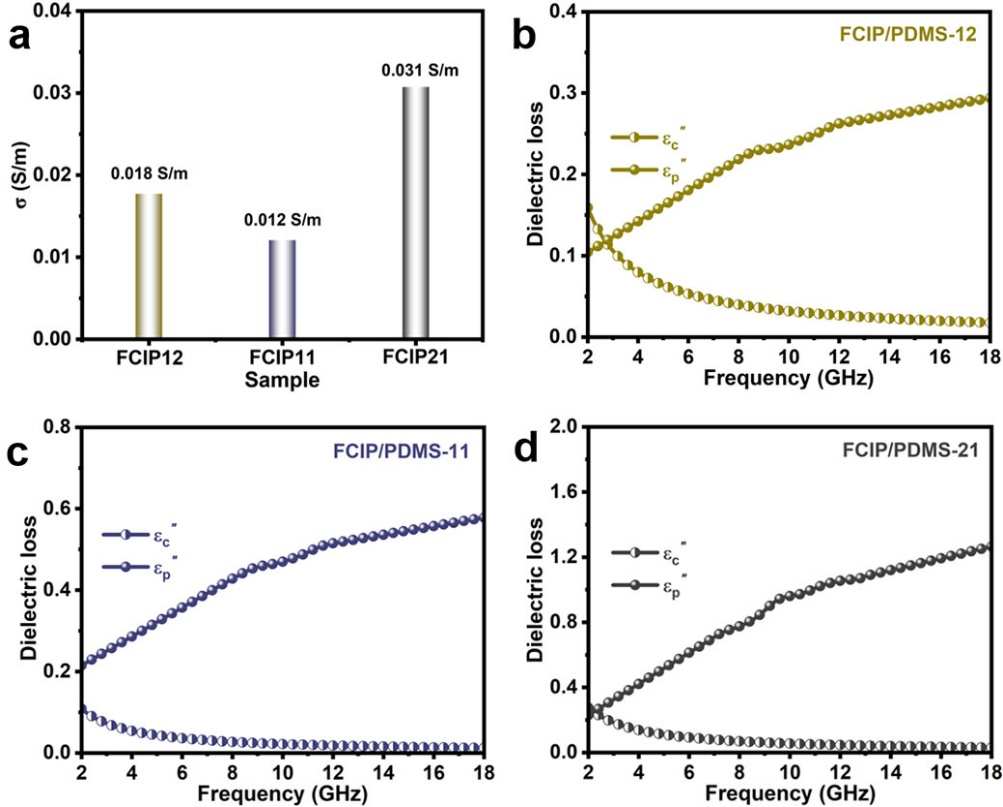
**

**Fig. S10 a** Conductivity of FCIP/PDMS. $\text{ε}_{\text{c}}"\sim f$ and $\text{ε}_{\text{p}}"\sim f$ curves of **b** FCIP/PDMS-12, **c** FCIP/PDMS-11, and **d** FCIP/PDMS-21

**
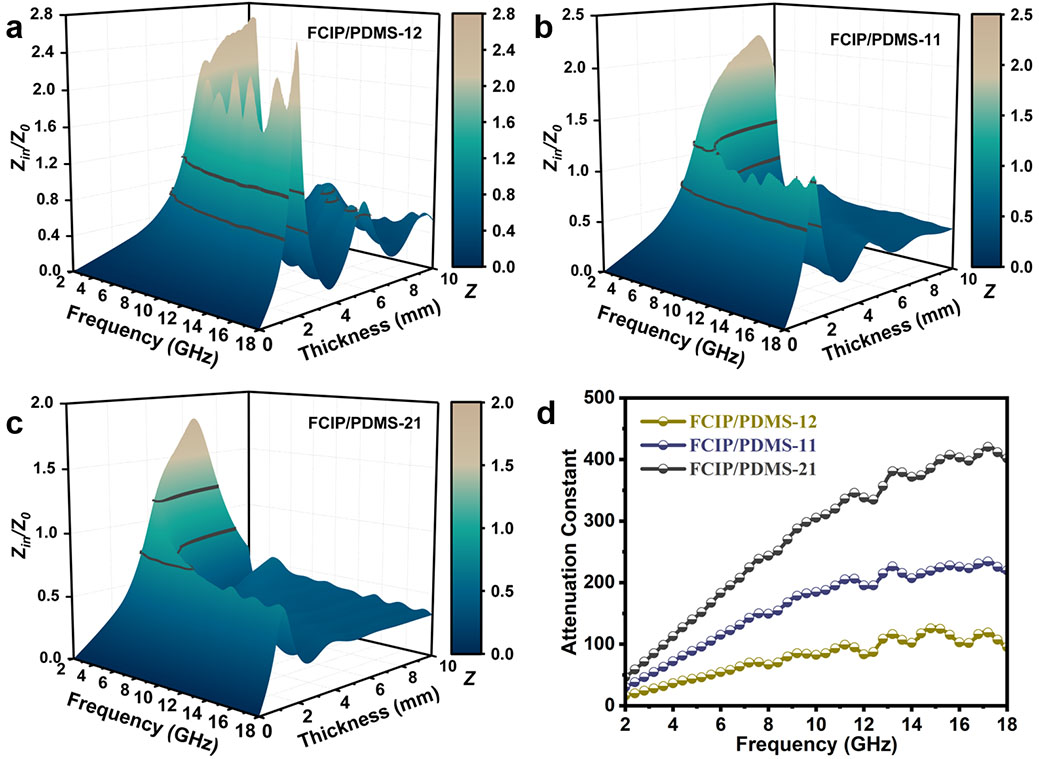
**

**Fig. S11** 3D Impedance matching color maps of **a** FCIP/PDMS-12, **b** FCIP/PDMS-11, and **c** FCIP/PDMS-21. **d** Attenuation constant of FCIP/PDMS

**
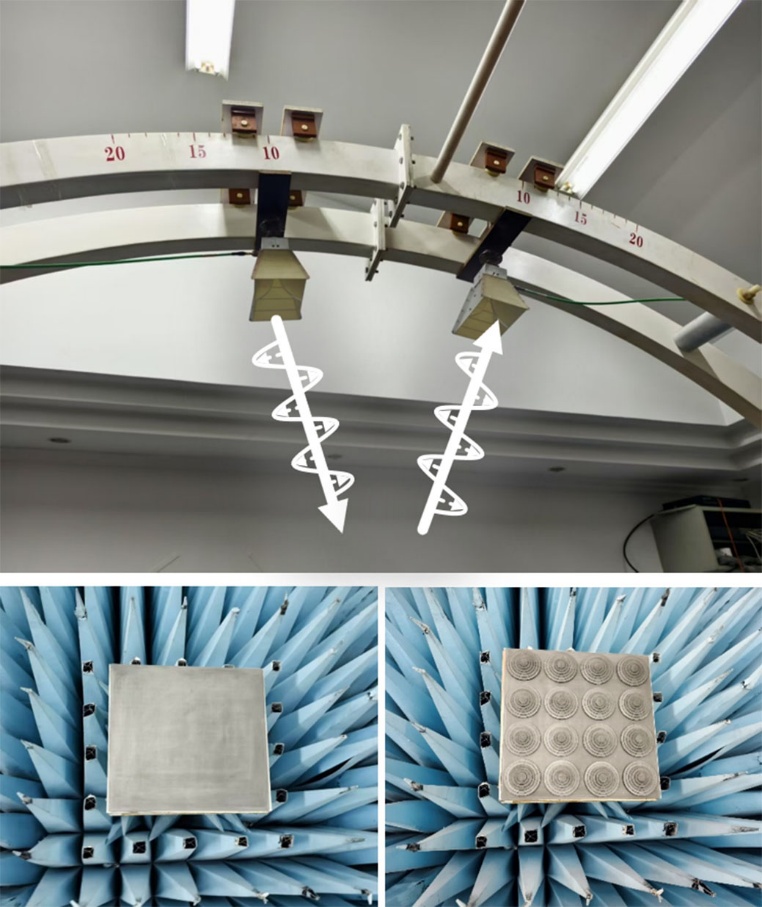
**

**Fig. S12** Schematic diagram of reflectance testing (NRL-arc method)


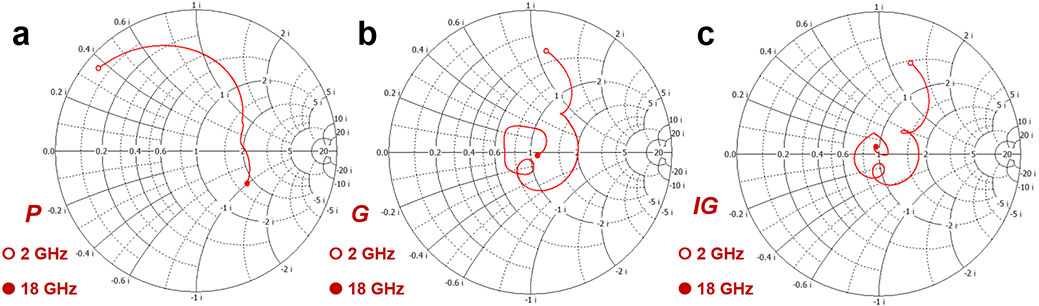


**Fig. S13** The Smith Chart of **a** P structures, **b** G structures, and **c** IG structures

**
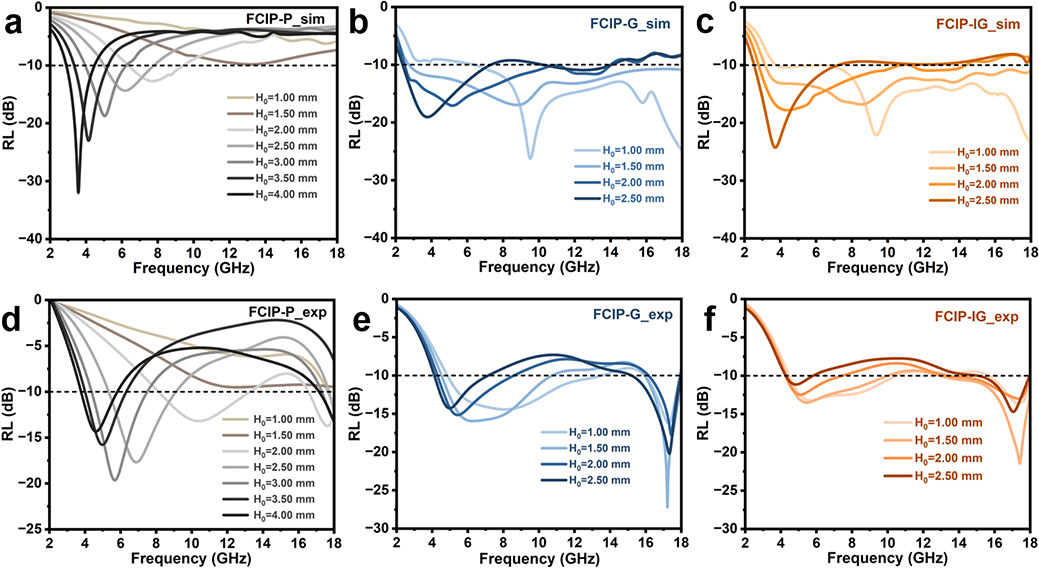
**

**Fig. S14** Simulated and experimental reflectance curves of **a, d** P, **b, e** G, and **c, f** IG structures under different H_0_ (H_1_= 2.00 mm)


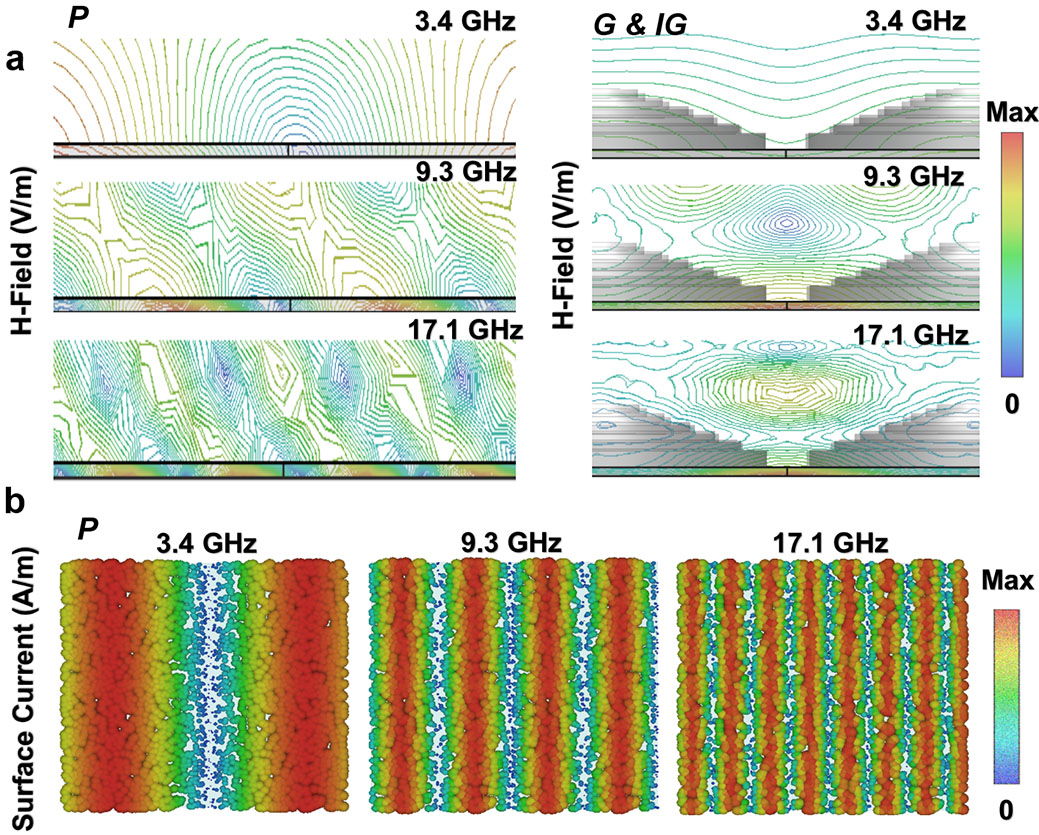


**Fig. S15** **a** Magnetic field distribution of P and IG devices. **b** Surface current distribution of IG devices


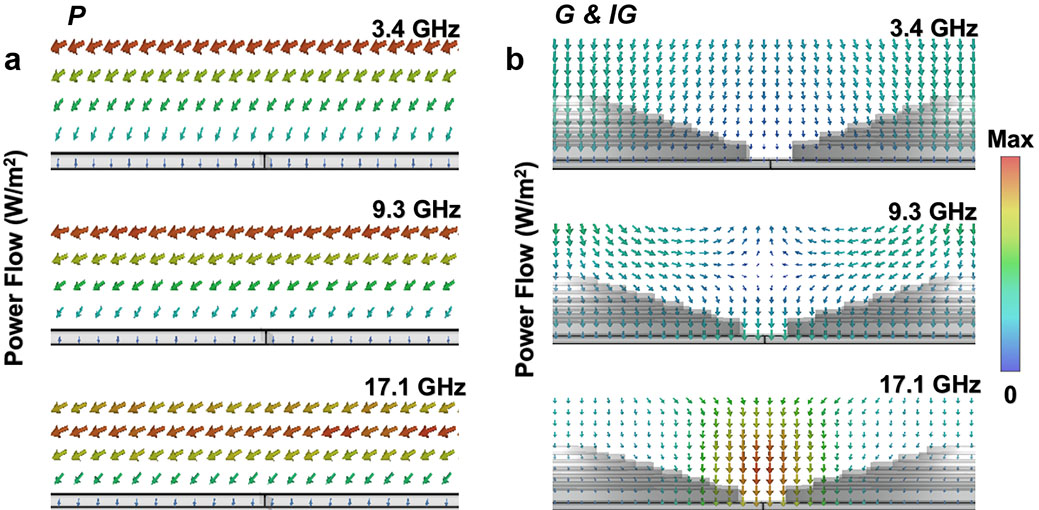


**Fig. S16** Power flow distribution of P and IG devices

**
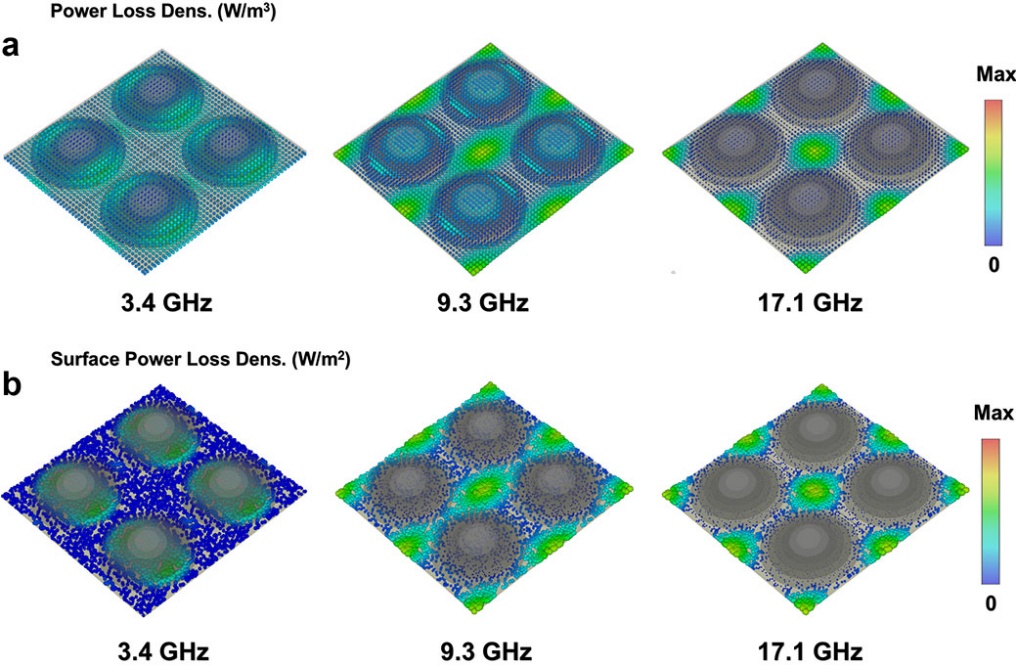
**

**Fig. S17 a** Power loss density and **b** Surface power loss density of IG devices

**
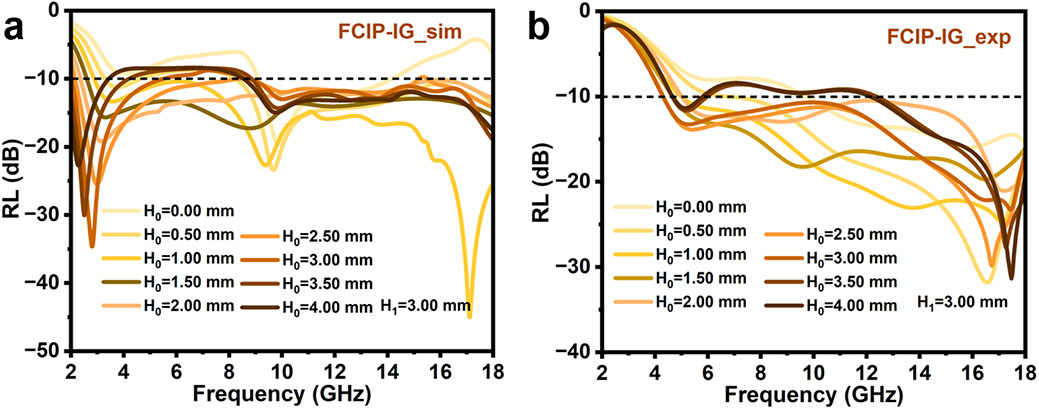
**

**Fig. S18 a, b** Simulated and experimental reflectance curves of IG devices under different H_0_ (H_1_= 3.00 mm)

**
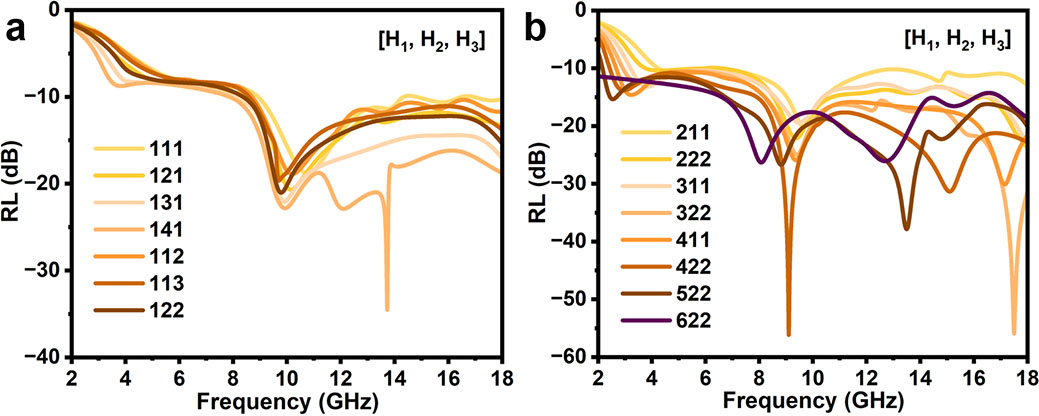
**

**Fig. S19 a, b** Original *RL-f* dataset for NN model construction


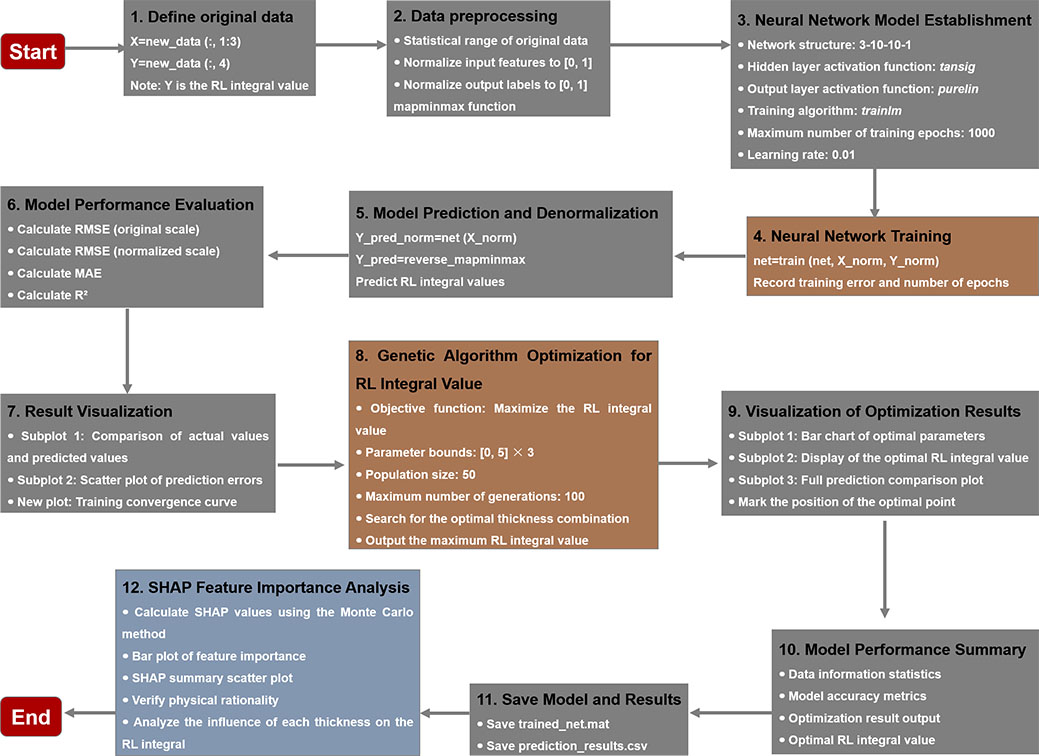


**Fig. S20** A flowchart of the data flow and training process


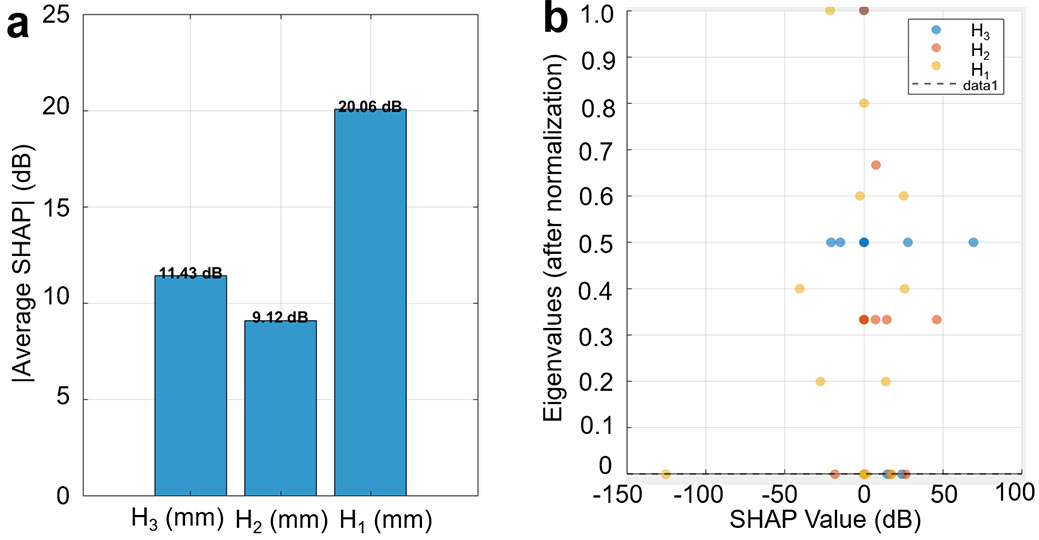


**Fig. S21 a** Feature importance analysis. b Direction of feature influence

**
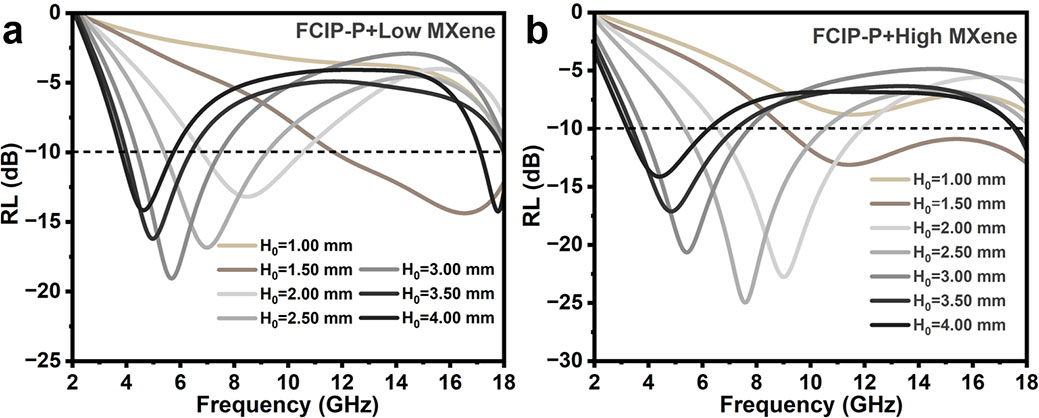
**

**Fig. S22** The *RL-f* curves of P structures coated with **a** Low MXene photochromic layers and **b** High MXene photochromic layers under different H_0_


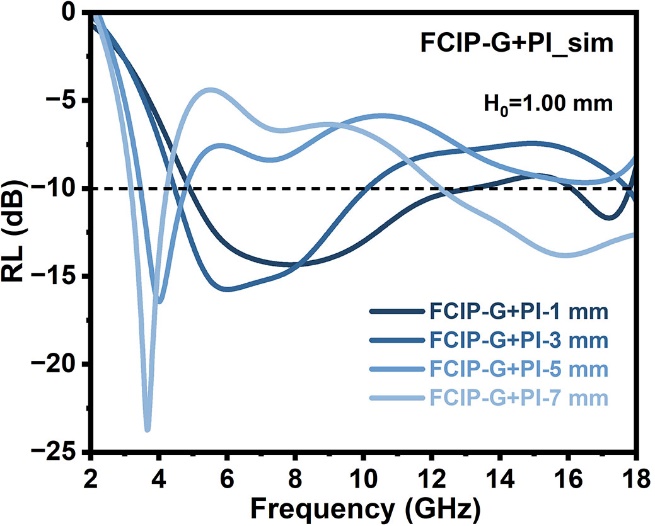


**Fig. S23** *RL-f* curves of the gradient (G) device loaded with PI of different thicknesses (1 mm, 3 mm, 5 mm, and 7 mm).

**
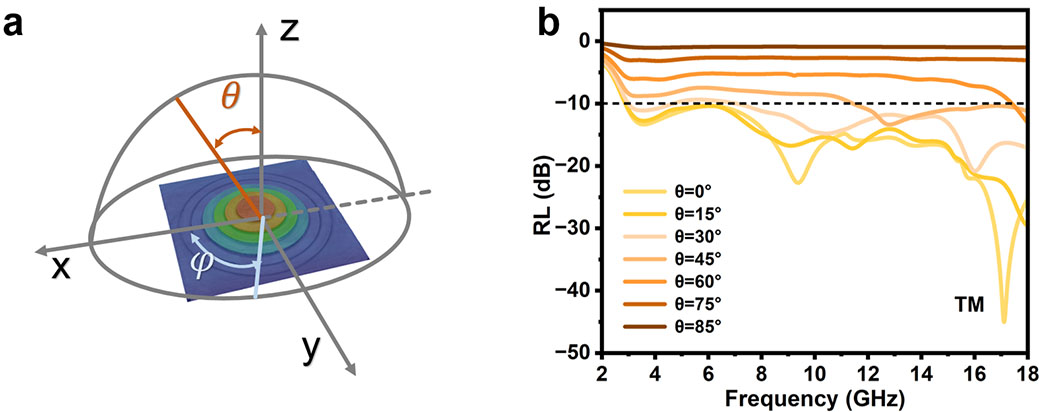
**

**Fig. S24** **a** Test diagram of IG devices at different incident angles ($\theta$) and polarization angles ($\varphi$). **b** The *RL-f* curves in TM modes at different $\theta$

**
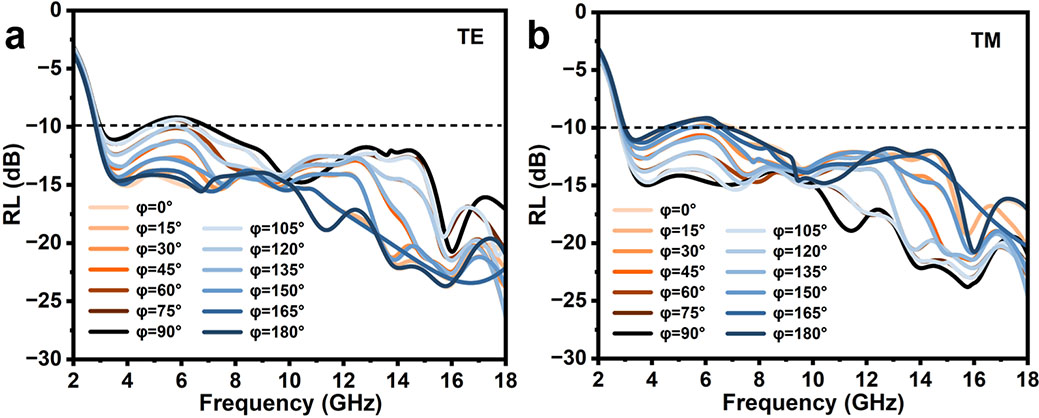
**

**Fig. S25** The *RL-f* curves in **a** TE modes and **b** TM modes at different $\varphi$


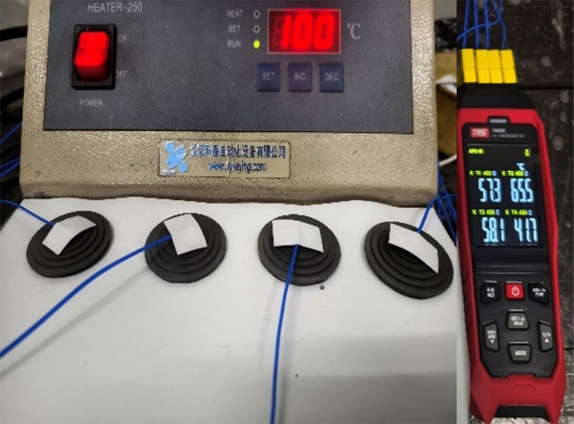


**Fig. S26** Schematic diagram of IR camera and thermocouple apparent temperature measurement device

**
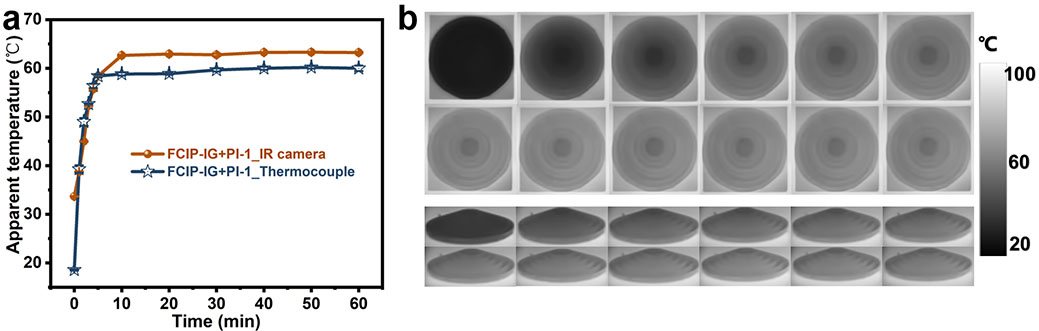
**

**Fig. S27 a** Apparent temperature curve of FCIP-IG+PI-1 under IR camera and thermocouple. **b** Thermal IR images of FCIP-IG+PI-1

**
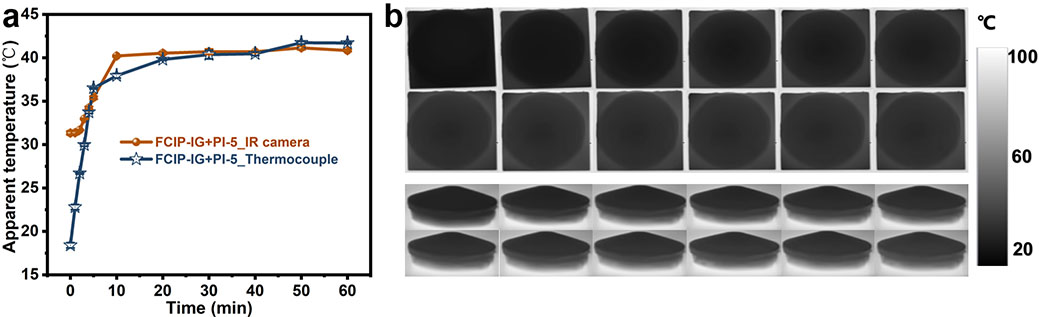
**

**Fig. S28 a** Apparent temperature curve of FCIP-IG+PI-5 under IR camera and thermocouple. **b** Thermal IR images of FCIP-IG+PI-5

**
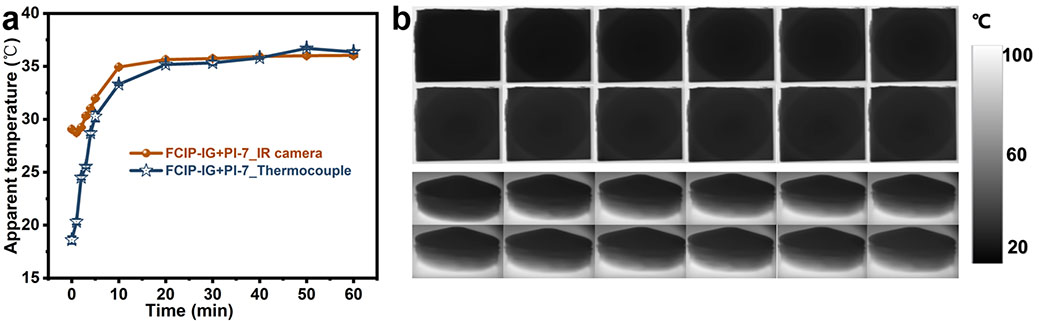
**

**Fig. S29 a** Apparent temperature curve of FCIP-IG+PI-7 under IR camera and thermocouple. **b** Thermal IR images of FCIP-IG+PI-7

**
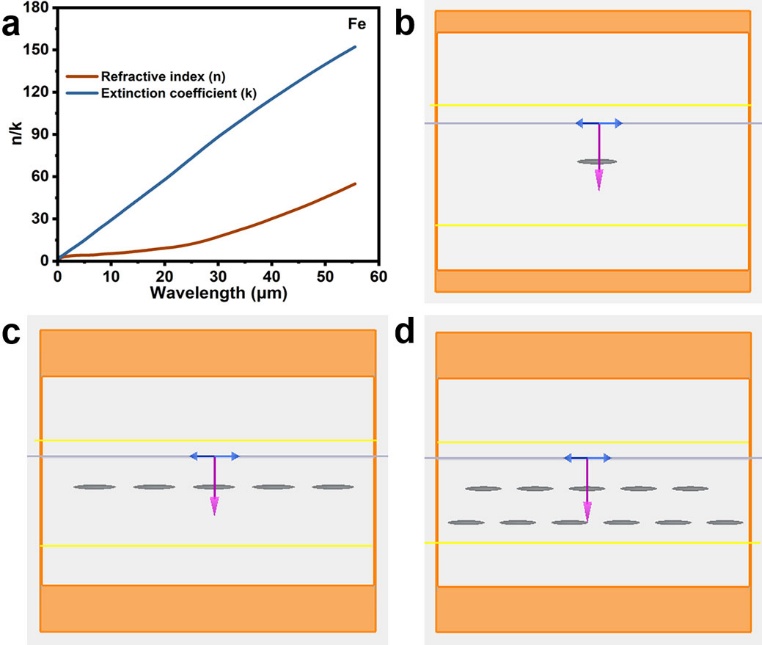
**

**Fig. S30 a** Optical parameters. **b** Optical simulation model

**
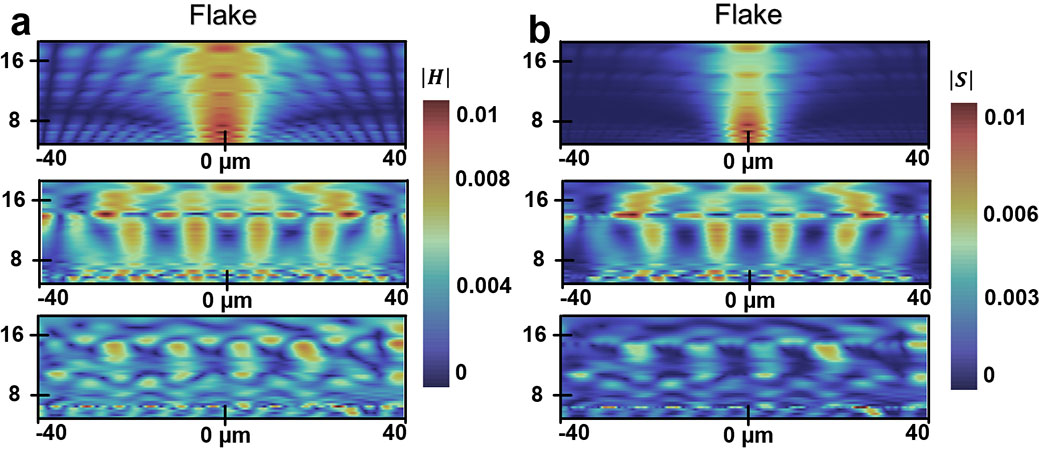
**

**Fig. S31** **a** Magnetic field distribution, **b** Power distribution of one, single-layer and multi-layer FCIP under optical simulation (FDTD)

**
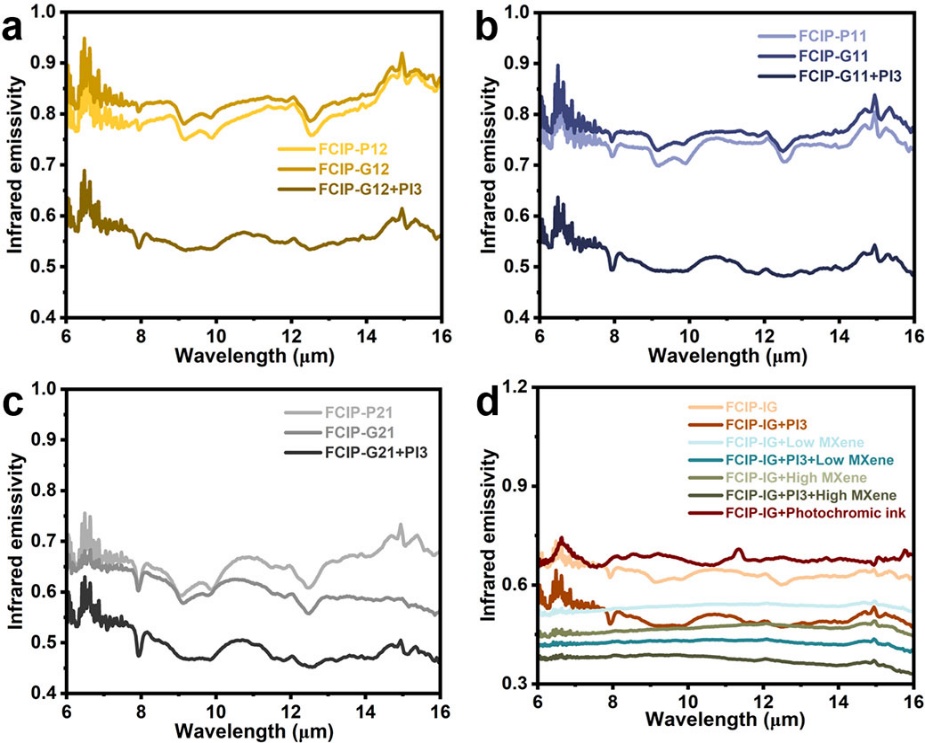
**

**Fig. S32** IR emissivity (8-14 μm) of **a** FCIP-P12, FCIP-G12, and FCIP-G12+PI-3, **b** FCIP-P11, FCIP-G11, and FCIP-G11+PI-3, **c** FCIP-P21, FCIP-G21, and FCIP-G21+PI-3, **d** FCIP-IG, FCIP-IG+PI-3, FCIP-IG+Low MXene, FCIP-IG+PI-3+Low MXene, FCIP-IG+High MXene, and FCIP-IG+PI-3+ High MXene, and FCIP-IG+Photochromic ink


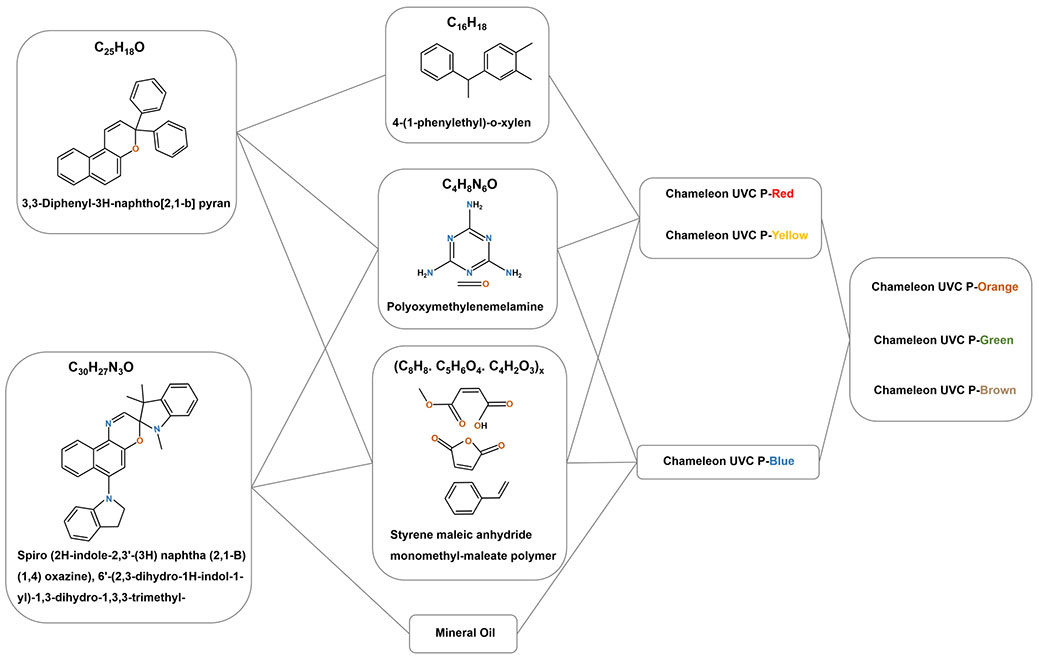


**Fig. S33** Chemical structural changes in photochromic inks

**
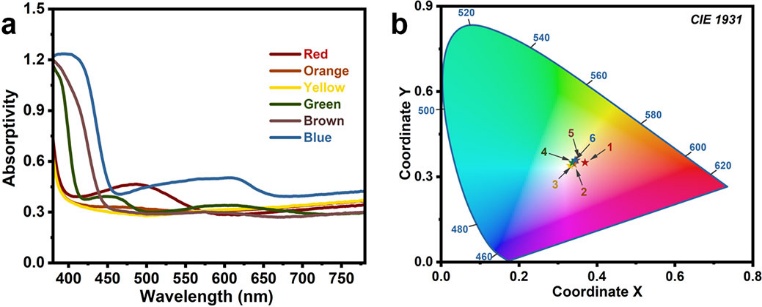
**

**Fig. S34 a** Absorptivity (380-780 nm) of different photochromic pigments (Red, Orange, Yellow, Green, Brown, and Blue). **b** CIE1931 color plot


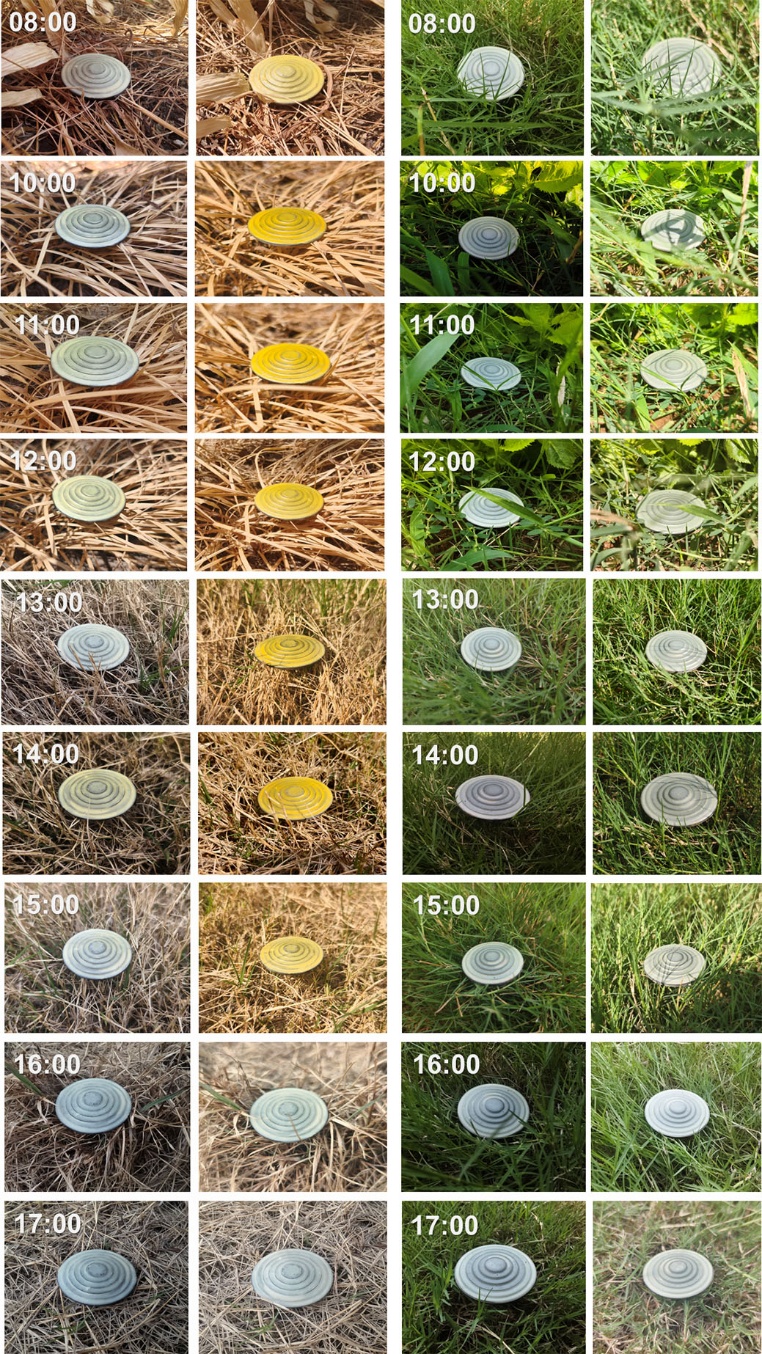


**Fig. S35** Schematic diagram showing the color change of the IG metadevice under weak and strong light illumination on a grass background (at different points throughout the day)


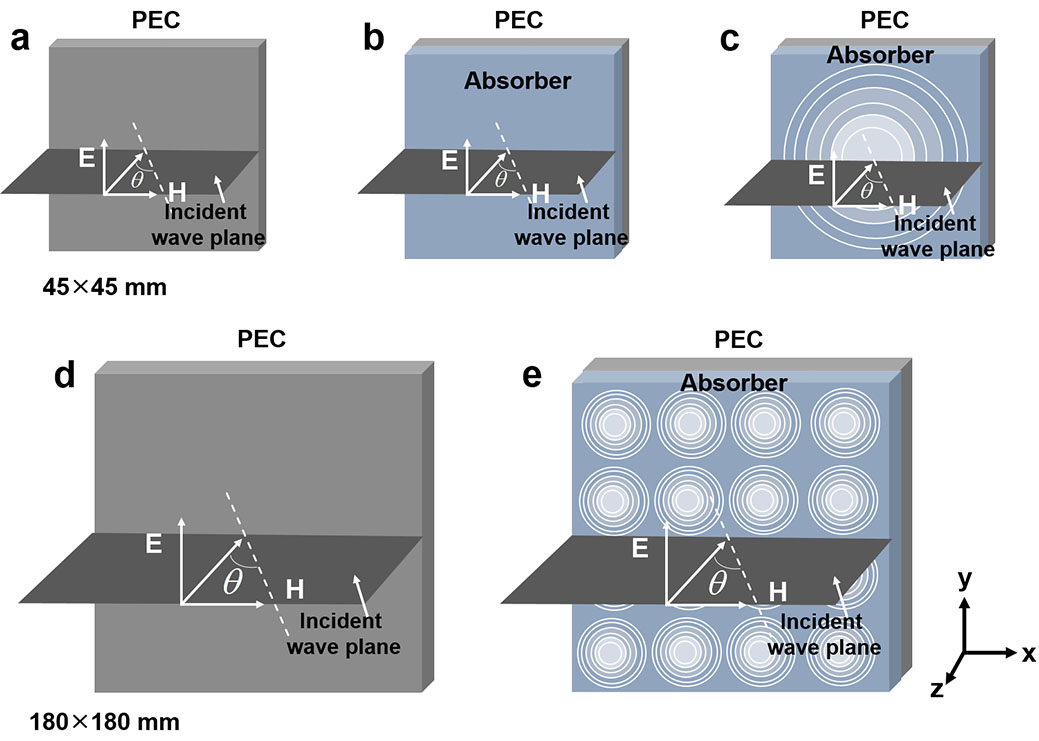


**Fig. S36** The RCS model (45 mm🞨45 mm) of **a** PEC, **b** P and **c** IG. The RCS model (180mm🞨180 mm) of **d** PEC and **e** IG

**
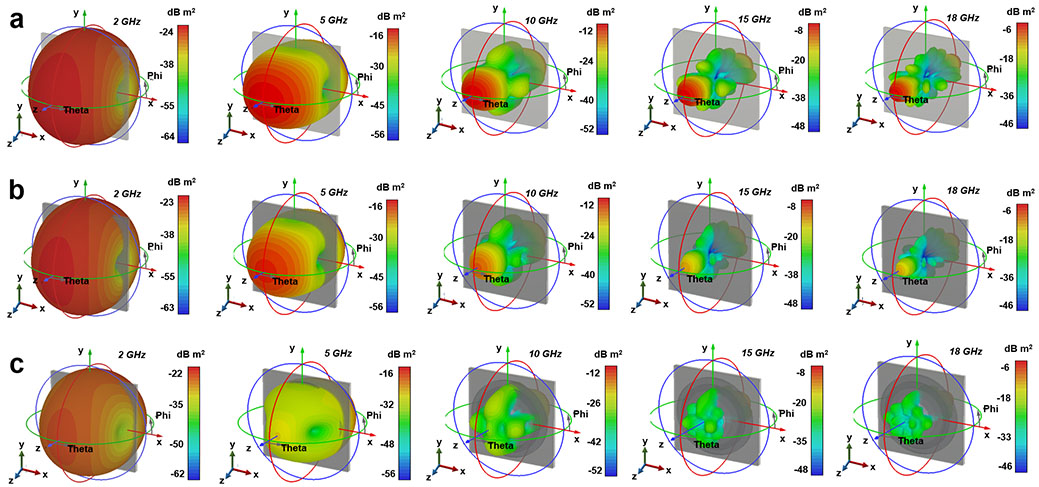
**

**Fig. S37** 3D radar scattering signals of **a** PEC, **b** FCIP-P, and **c** FCIP-IG

**
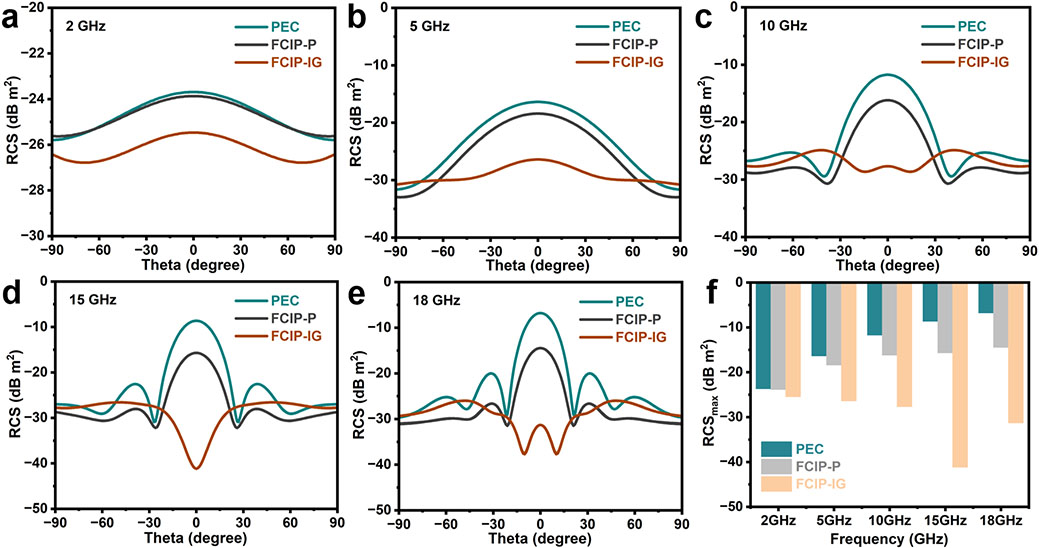
**

**Fig. S38** **a-e** RCS simulated curves of PEC, FCIP-P, and FCIP-IG. **f** Statistical graph for RCS_max_


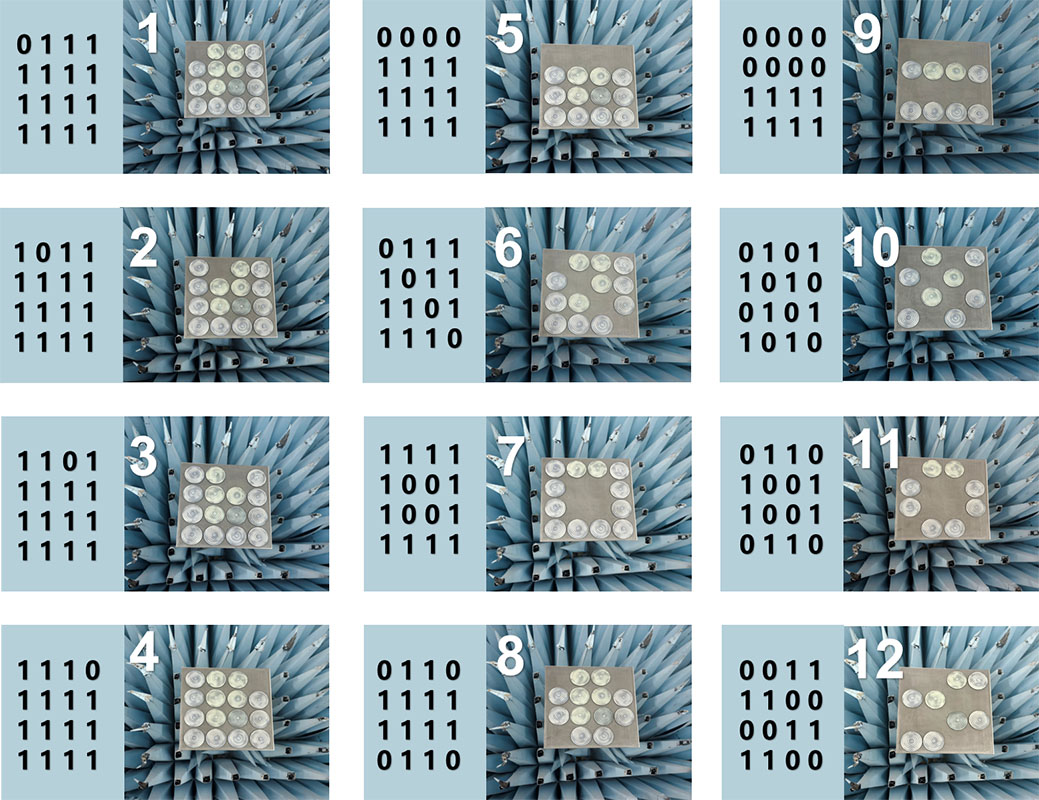


**Fig. S39** Schematic diagram of 12 pattern

**
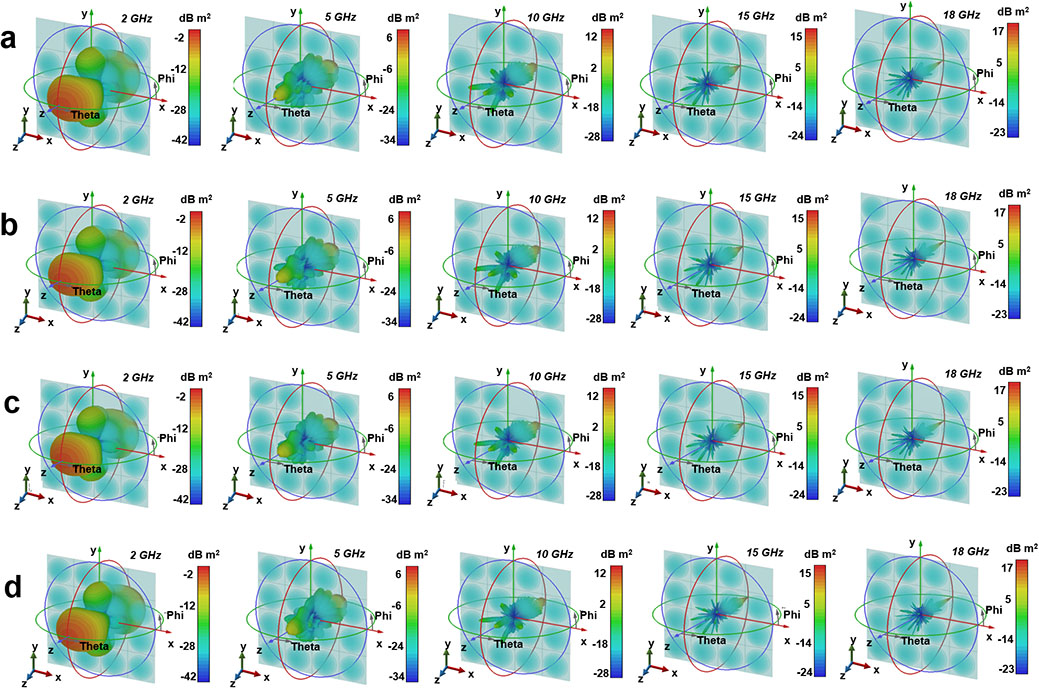
**

**Fig. S40** 3D radar scattering signals of **a** Pattern 1, **b** Pattern 2, **c** Pattern 3, and **d** Pattern 4

**
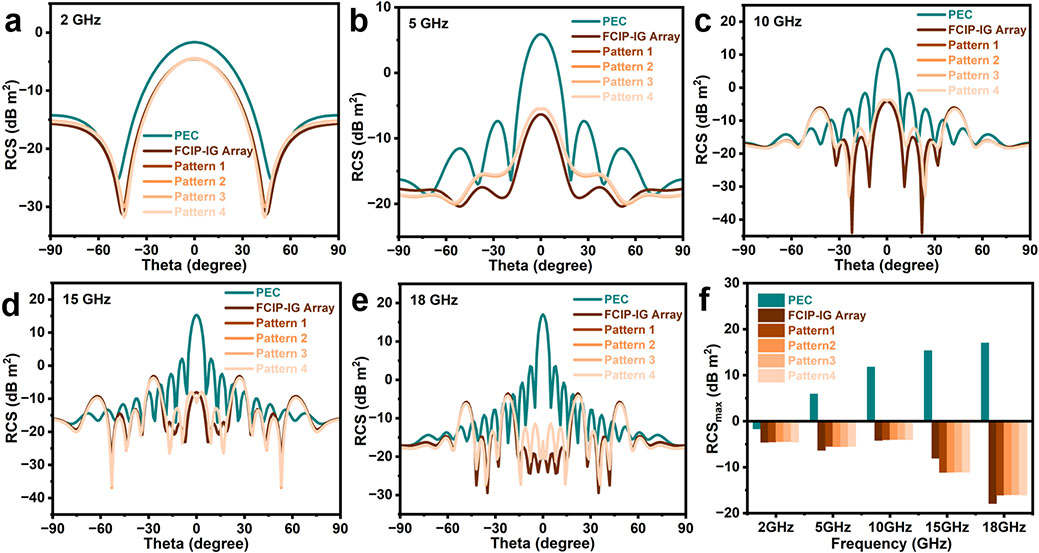
**

**Fig. S41** **a-e** RCS simulated curves of PEC-180 mm, FCIP-IG Array, Pattern 1, Pattern 2, Pattern 3, and Pattern 4. **f** Statistical graph for RCS_max_

**
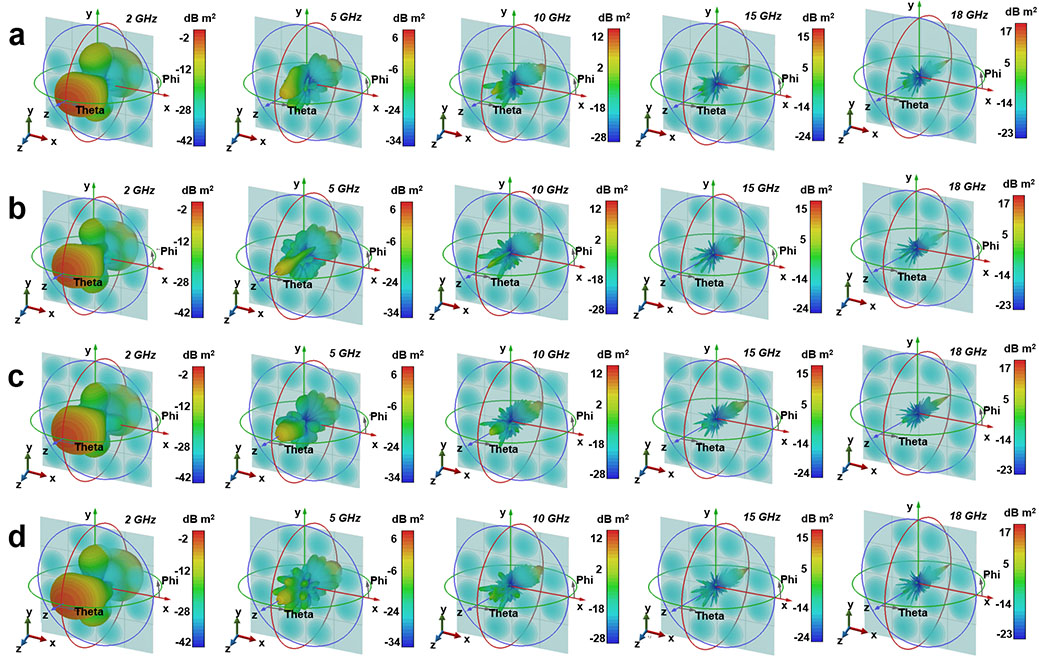
**

**Fig. S42** 3D radar scattering signals of **a** Pattern 5, **b** Pattern 6, **c** Pattern 7, and **d** Pattern 8

**
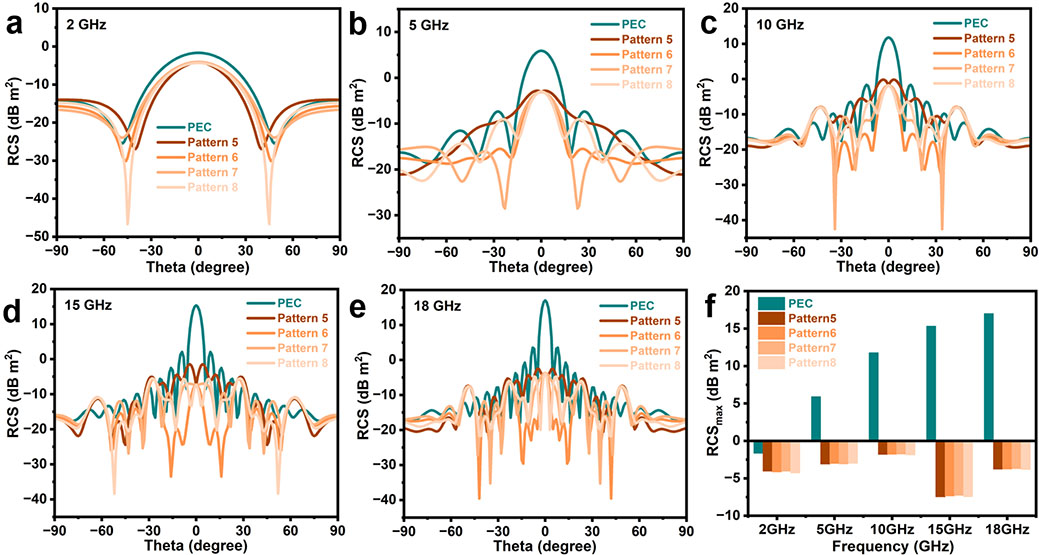
**

**Fig. S43** **a-e** RCS simulated curves of PEC-180 mm, Pattern 5, Pattern 6, Pattern 7, and Pattern 8. **f** Statistical graph for RCS_max_

**
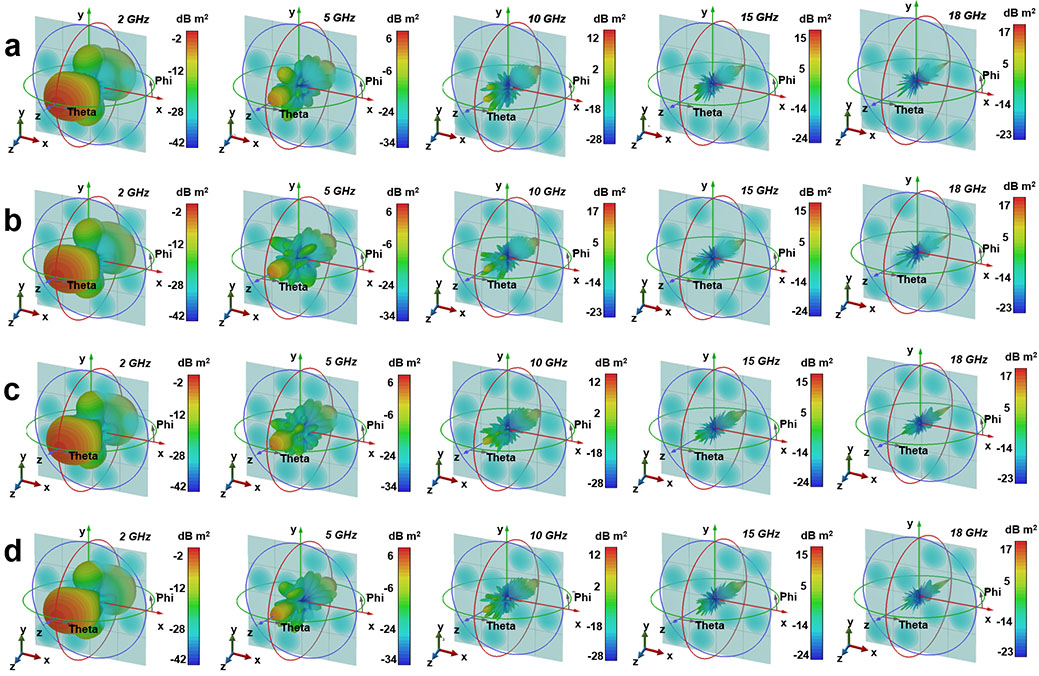
**

**Fig. S44** 3D radar scattering signals of **a** Pattern 9, **b** Pattern 10, **c** Pattern 11, and **d** Pattern 12

**
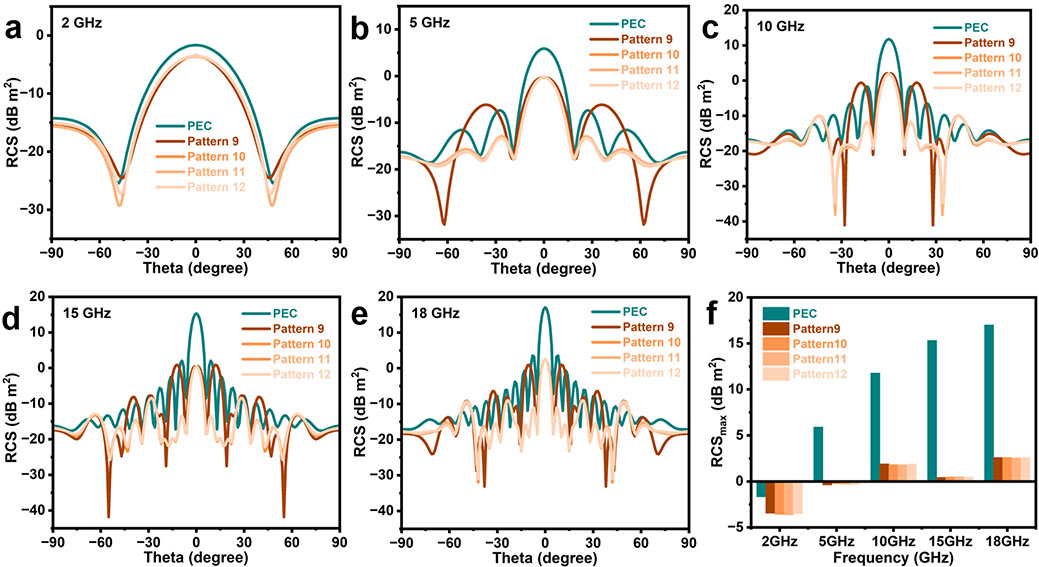
**

**Fig. S45** **a-e** RCS simulated curves of PEC-180 mm, Pattern 9, Pattern 10, Pattern 11, and Pattern 12. **f** Statistical graph for RCS_max_

**
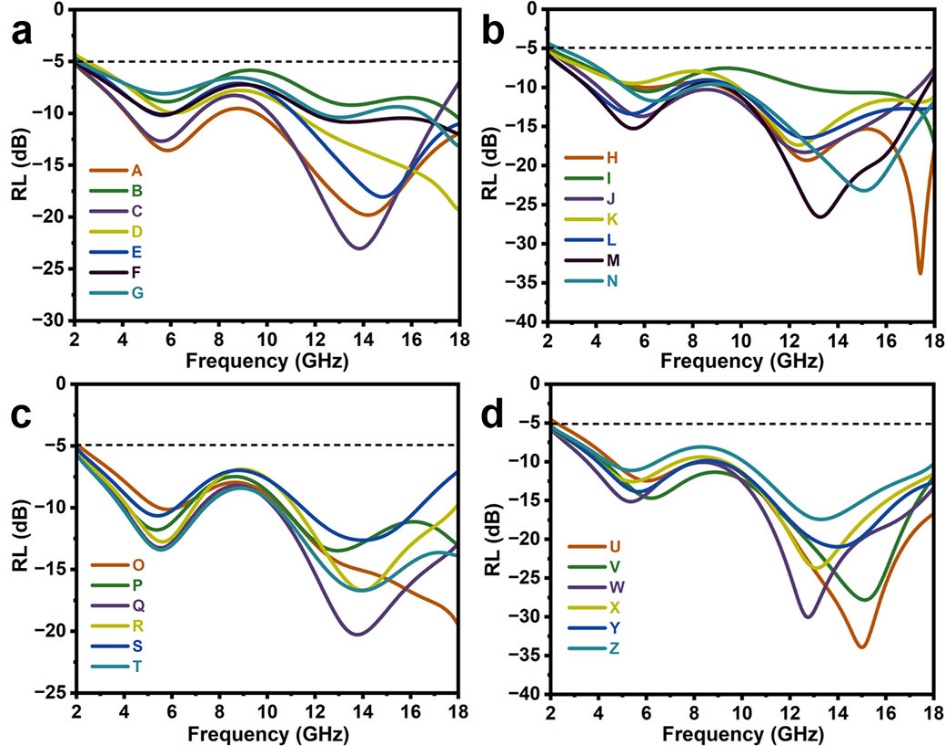
**

**Fig. S46** **a-d** The *RL-f* curves of pattern A-Z


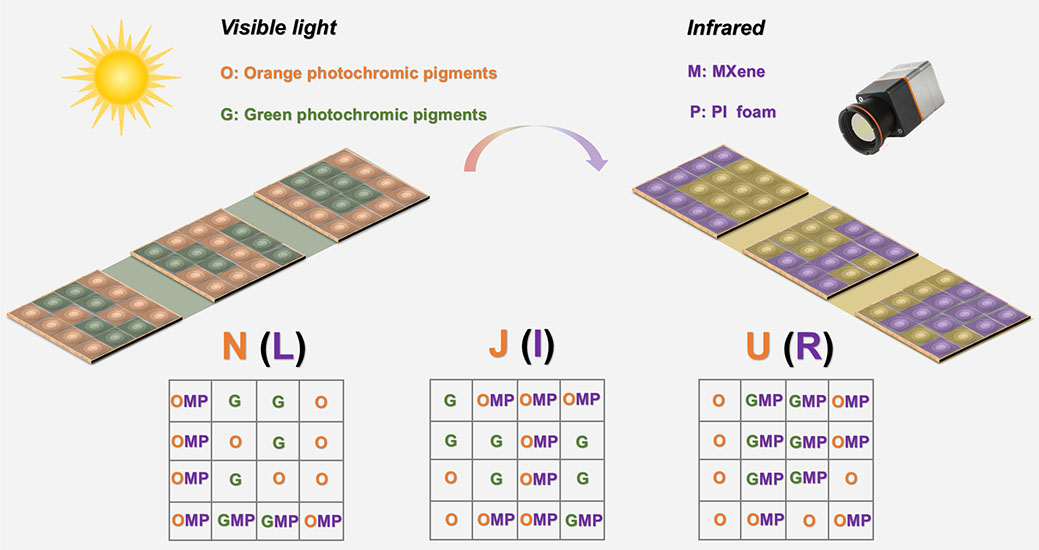


**Fig. S47** Detailed schematic diagram of switching between VIS and IR encoding/decoding modes

**
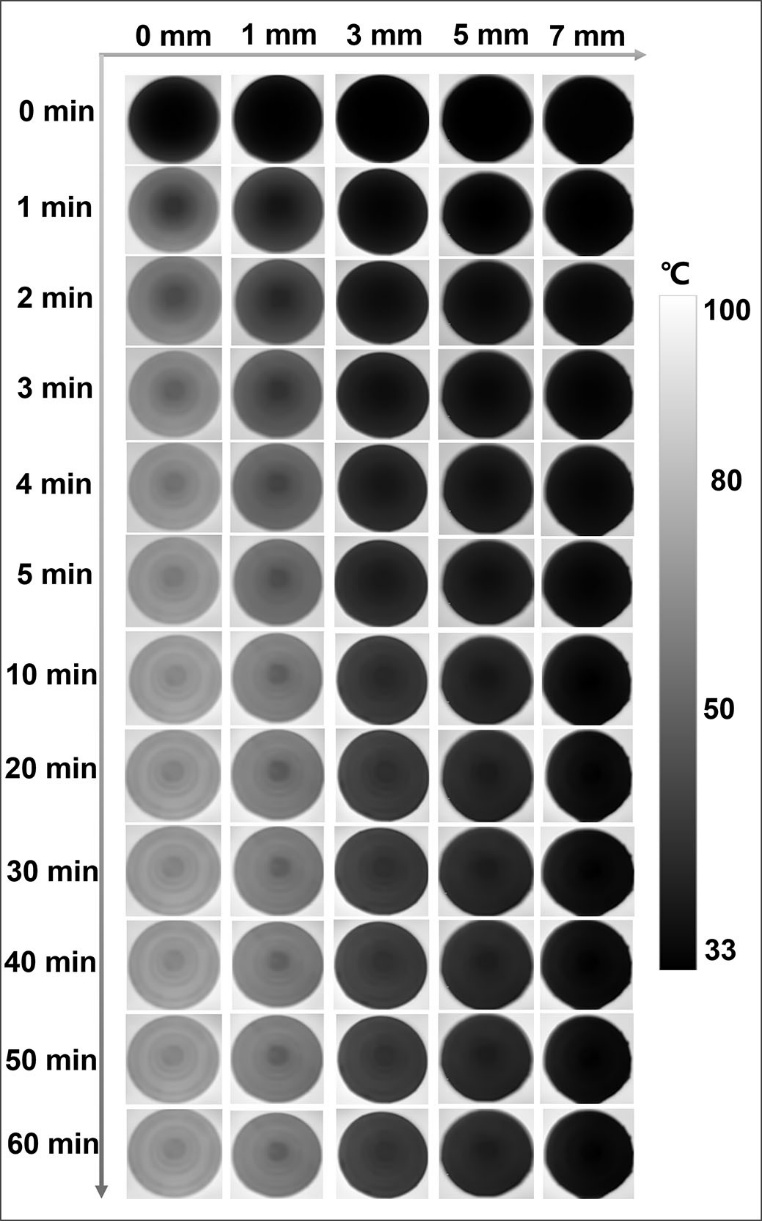
**

**Fig. S48** Thermal IR images of IG, IG+PI-1, IG+PI-3, IG+PI-5, and IG+PI-7 after three months

**
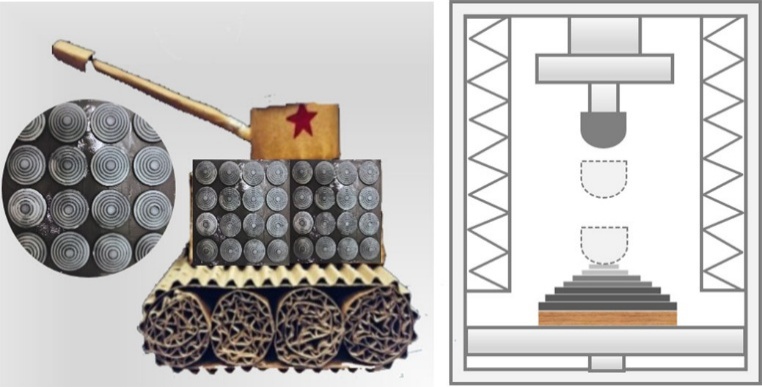
**

**Fig. S49** Schematic diagram of drop weight impact test apparatus


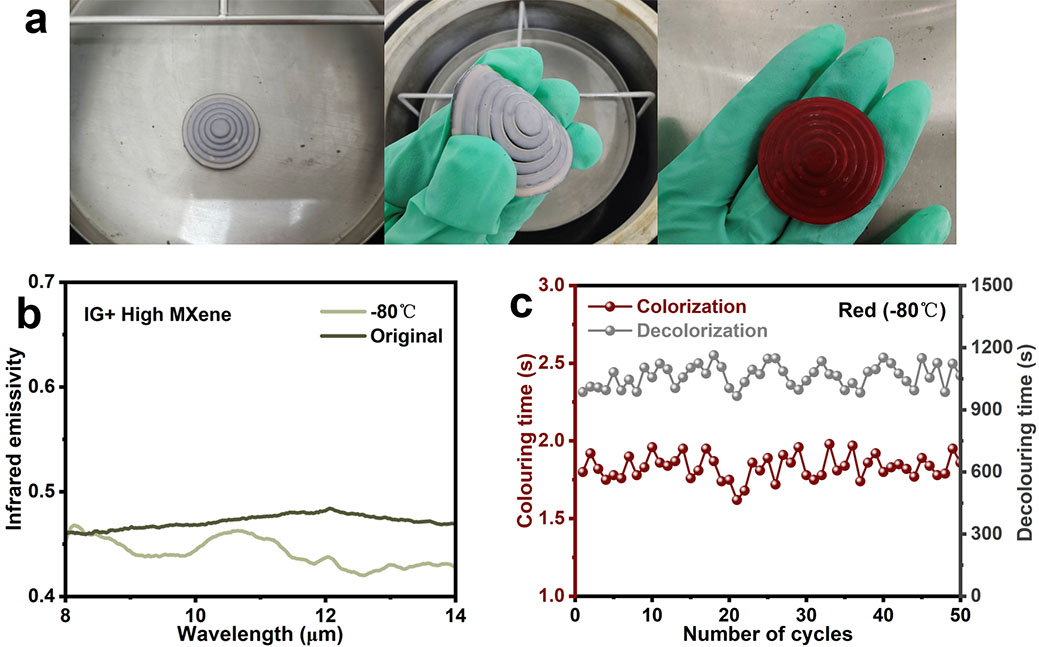


**Fig. S50 a** Anti-icing properties of devices at low temperatures. **b** IR emissivity (8-14 μm). **c** Coloring and decoloring times of the red photochromic ink within 50 cycles of the device


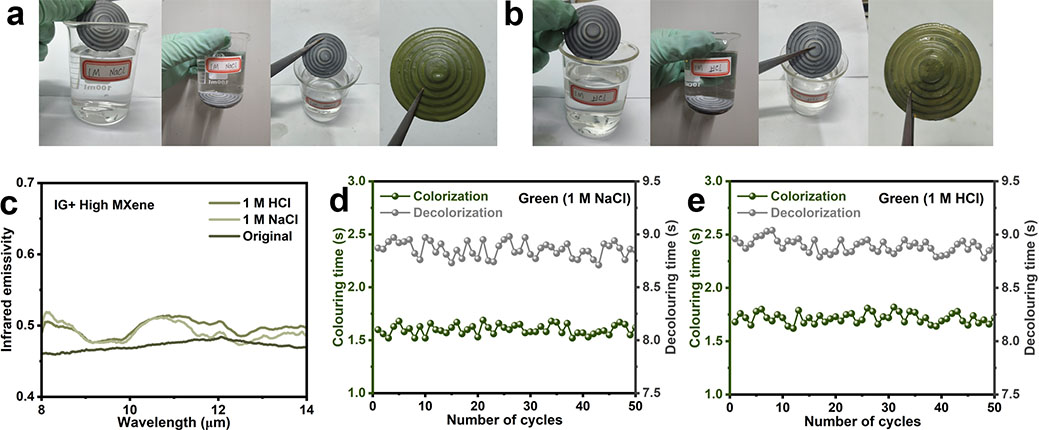


**Fig. S51 a, b** Salt resistance (1M NaCl, 2h) and acid resistance (1M HCl, 2h) test. **c** IR emissivity (8-14 μm). **d, e** Coloring and decoloring times of the green photochromic ink within 50 cycles of the device


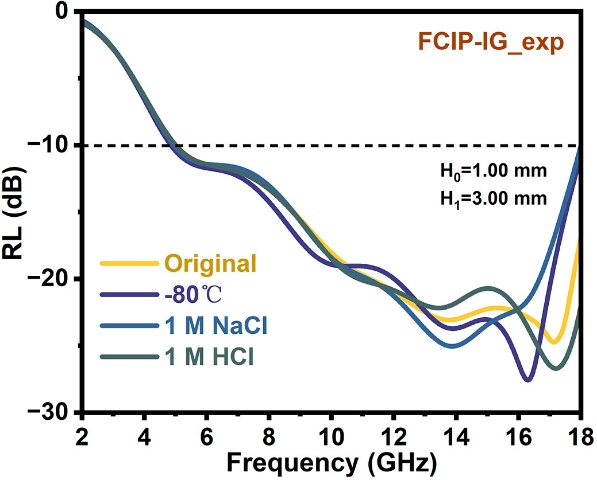


**Fig. S52** *RL-f* curves of the device after treatment under extreme environments (-80 °C, 1 M NaCl, 1 M HCl)


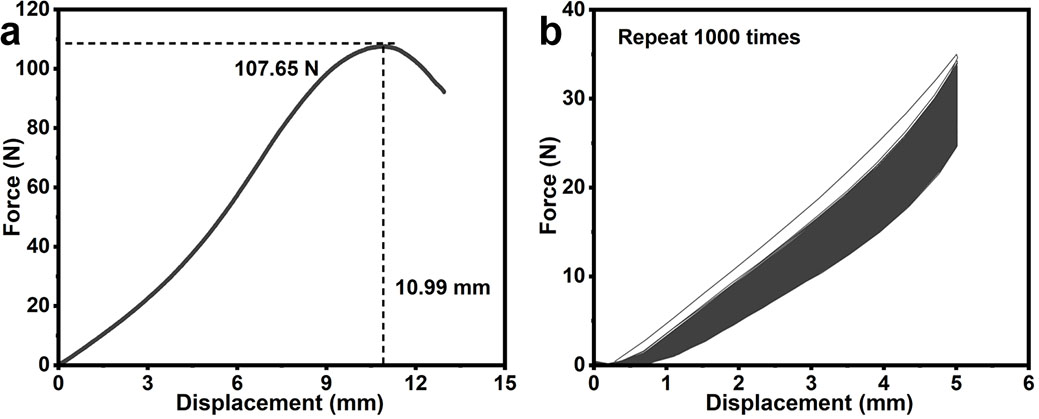


**Fig. S53 a** Force-displacement curve of the IG unit under single three-point bending. **b** Force-displacement curves of the IG unit under 1000 cycles of three-point bending

**Supplementary Videos**

**Video S1** The thermal camouflage effect of the IG+PI-1, IG+PI-3, IG+PI-5, and IG+PI-7 evolves over time (top).

**Video S2** The thermal camouflage effect of the IG+PI-1, IG+PI-3, IG+PI-5, and IG+PI-7 evolves over time (side).

**Video S3** IG metadevice color change demonstration process (red, orange, yellow, green, brown, and blue).

**Video S4** The thermal camouflage effect of the IG+PI-1, IG+PI-3, IG+PI-5, and IG+PI-7 evolves over time (top) after three months.

**Video S5** IG metadevice color change demonstration process (red, orange, yellow, green, brown, and blue) after three months.

**Supplementary References**

1. Q. Wang, X.J. Liu, J. Cui, Y.H. Yan, Gradient and flexible poly (vinylidene fluoride) composite with synergistically enhanced wave absorption performance by MXene and hollow cobalt ferrite. Chem. Eng. J. **520**, 165961 (2025). <https://doi.org/10.1016/j.cej.2025.165961>
2. A. Biswas, S.C. Wang, C.L. Milne, G.A. Alvarez, M.A. Islam et al., Carbon doped boron nitride nano-coatings for durable, low emissivity glass windows. Adv. Mater. **37**, 2507557 (2025). <https://doi.org/10.1002/adma.202507557>
3. Y. Jia, D.Q. Liu, D.S. Chen, Y.Z. Jin, Y.F. Ge et al., Realizing sunlight-induced efficiently dynamic infrared emissivity, modulation based on aluminum-doped zinc oxide nanocrystals. Adv. Sci. **11**, 2405962 (2024). <https://doi.org/10.1002/advs.202405962>
4. Y. Jia, D.Q. Liu, D.S. Chen, Y.Z. Jin, C. Chen et al., Transparent dynamic infrared emissivity regulators. Nat. Commun. **14**, 5087 (2023). <https://doi.org/10.1038/s41467-023-40902-w>
5. W. Wu, L.P. Tong, H. Zhou, T.X. Fan, Combined experimental and DFT study on 2D MoSe toward low infrared emissivity. Adv. Funct. Mater. **32**, 2201906 (2022). <https://doi.org/10.1002/adfm.202201906>
6. H.L. Zhang, X. Zhang, W.H. Sun, M.J. Chen, Y.J. Xiao et al., All-solid-state transparent variable infrared emissivity devices for multi-mode smart windows. Adv. Funct. Mater. **34**, 2307356 (2024). <https://doi.org/10.1002/adfm.202307356>
7. Z.T. Li, Y.J. Xiao, X. Zhang, B. Sun, H.L. Zhang et al., A novel transparent memristor-type infrared emissivity modulator for multispectral compatible display. Small Struct. **5**, 2400030 (2024). <https://doi.org/10.1002/sstr.202400030>
8. X.P. Jiang, J. Nong, X. Li, X.Y. Liao, J.X. Zeng et al., Laser-adaptive inverse-design metamaterials for durable regulation from visible-infrared-LiDAR compatible camouflage to optical limiter. Laser Photonics Rev. **19**, e00881 (2025). <https://doi.org/10.1002/lpor.202500881>
9. Y.H. Jin, J.Y. Cheng, S. Jiang, X.J. Zou, Y.P. Wang et al., Conductance reinforced relaxation attenuation with strong metal-N coordination in multivariate π-conjugated MOFs for integrated radar-infrared camouflage. Adv. Mater. **37**, 2501330 (2025). <https://doi.org/10.1002/adma.202501330>
10. X.Y. Xu, B. Yu, N.R. Vokhidova, Y.X. Lu, Multispectral-stealth machine dog cladding: AlPO/NiFe@CNT composites enabling concurrent flame retardancy and Radar-IR-THz compatible invisibility. Chem. Eng. J. **515**, 163512 (2025). <https://doi.org/10.1016/j.cej.2025.163512>
11. P. Wang, W.R. Xie, J. Zhang, Y. Sun, S.Y. Huang et al., Dual-functional photonic battery enabling dynamic radiative thermal management and power supply. Adv. Mater. **37**, 2412328 (2025). <https://doi.org/10.1002/adma.202412328>
12. Y.W. Zhang, G.H. Li, S.P. Ma, Z. Li, F. Fan et al., Switchable multi-spectral electromagnetic defense in the ultraviolet, visible, infrared, gigahertz, and terahertz bands using a magnetically-controllable soft actuator. ACS Nano **19**, 11295-11308 (2025). <https://doi.org/10.1021/acsnano.5c00138>
13. J. Zhu, Y. Hu, J.H. Hu, B. Zhou, Y.H. Ye et al., Bioinspired multilayer composites based on nano-self-assembled thermomechanical expressway for efficient thermal management. Small **21**, 2412452 (2025). <https://doi.org/10.1002/smll.202412452>
14. Y.P. Li, S.Z. Ding, Z.M. An, T.T. Xu, R.B. Zhang, Ultra-thin compatible stealth metacoating: Graded control of radar and infrared waves under long-term high temperatures. Compos. Part B-Eng. **297**, 112295 (2025). <https://doi.org/10.1016/j.compositesb.2025.112295>
15. H.L. Peng, B. Cai, Y. Zhang, L.C. Gao, P.Y. Zhao, et al., Radar-terahertz-infrared compatible stealth coaxial silver nanowire@carbon nano-cable aerogel. Angew. Chem. Int. Edit. **64**, e202421090 (2025). <https://doi.org/10.1002/anie.202421090>
16. Z.Y. Li, Y. Xu, L.H. Wu, Y. Sun, M.N. Zhang et al., Carbon nanocage-in-microcage structure with tunable carbon-coated nickel as a microwave absorber with infrared stealth property. Adv. Sci. **12**, 2412890 (2025). <https://doi.org/10.1002/advs.202412890>
17. W.H. Gu, A.L. Xia, C.G. Jin, H.Y. Zhang, H.L. Li et al., An ultralight, eco-friendly 3D porous carbon aerogel derived from cotton nano-cellulose for infrared stealth and microwave absorption. Carbon **229**, 119565 (2024). <https://doi.org/10.1016/j.carbon.2024.119565>
18. B.B. Yan, S.Q. Liu, Y. Yuan, X.N. Hou, M. Zhou et al., Polymer-regulating MXene@dopamine electroactive gel-inks for textile-based multi-protective wearables. Adv. Funct. Mater. **34**, 2401097 (2024). <https://doi.org/10.1002/adfm.202401097>
19. X.Y. Ye, Y. Chen, J. Yang, H.Y. Yang, D.W. Wang et al., Sustainable wearable infrared shielding bamboo fiber fabrics loaded with antimony doped tin oxide/silver binary nanoparticles. Adv. Compos. Hybrid Mater. **6**, 106 (2023). <https://doi.org/10.1007/s42114-023-00683-8>
20. M. Zhao, H. Zhu, B. Qin, R. Zhu, J. Zhang et al., High-temperature stealth across multi-infrared and microwave bands with efficient radiative thermal management. Nano-Micro Lett. **17**, 199 (2025). <https://doi.org/10.1007/s40820-025-01712-5>
21. Y.Q. Xiong, Y.T. Zhou, J.L. Tian, W.L. Wang, W. Zhang et al., Scalable, color-matched, flexible plasmonic film for visible-infrared compatible camouflage. Adv. Sci. **10**, 2303452 (2023). <https://doi.org/10.1002/advs.202303452>
22. X. Chen, Y.L. Li, S.Y. Cheng, K.J. Wu, Q. Wang et al., Liquid metal-MXene-based hierarchical aerogel with radar-infrared compatible camouflage. Adv. Funct. Mater. **34**, 2308274 (2024). <https://doi.org/10.1002/adfm.202308274>
23. M.Y. Pan, Y. Huang, Q. Li, H. Luo, H.Z. Zhu et al., Multi-band middle-infrared-compatible camouflage with thermal management via simple photonic structures. Nano Energy **69**, 104449 (2020). <https://doi.org/10.1016/j.nanoen.2020.104449>
24. J. Wang, Z.X. Wu, X.Y. Sun, Z.Q. Tang, C. Wang et al., Multi-band compatible camouflage enabled by phase transition modulation of flexible GST films. Chem. Eng. J. **499**, 156128 (2024). <https://doi.org/10.1016/j.cej.2024.156128>
25. T. Zhang, Y.P. Duan, J.Y. Liu, H. Lei, J.X. Sun et al., Asymmetric electric field distribution enhanced hierarchical metamaterials for radar-infrared compatible camouflage. J. Mater. Sci. Technol. **146**, 10-18 (2023). <https://doi.org/10.1016/j.jmst.2022.10.043>
26. J.K. Huang, Y.T. Wang, L.M. Yuan, C. Huang, J.M. Liao et al., Large-area and fexible plasmonic metasurface for laser-infrared compatible camouflage. Laser Photonics Rev. **17**, 2200616 (2023). <https://doi.org/10.1002/lpor.202200616>
27. X.D. Feng, M.B. Pu, F. Zhang, R. Pan, S. Wang et al., Large-area low-cost multiscale-hierarchical metasurfaces for multispectral compatible camouflage of dual-band lasers, infrared and microwave. Adv. Funct. Mater. **32**, 2205547 (2022). <https://doi.org/10.1002/adfm.202205547>
28. L.M. Yuan, C. Huang, J.M. Liao, C. Ji, J.K. Huang et al., A dynamic thermal camouflage metadevice with microwave scattering reduction. Adv. Sci. **9**, 2201054 (2022). <https://doi.org/10.1002/advs.202201054>
29. J. Nam, I. Chang, J.S. Lim, H. Woo, J.G. Yook et al., Flexible metasurface for microwave-infrared compatible camouflage via particle swarm optimization algorithm. Small **19**, 2302848 (2023). <https://doi.org/10.1002/smll.202302848>
30. Y.Z. Wang, H.L. Luo, Y.Z. Shao, H. Wang, T. Liu et al., Detection and anti-detection with microwave-infrared compatible camouflage using asymmetric composite metasurface. Adv. Sci. **11**, 2410364 (2024). <https://doi.org/10.1002/advs.202410364>
31. W. Xi, Y.J. Lee, S.L. Yu, Z.H. Chen, J. Shiomi et al., Ultrahigh-efficient material informatics inverse design of thermal metamaterials for visible-infrared-compatible camouflage. Nat. Commun. **14**, 4694 (2023). <https://doi.org/10.1038/s41467-023-40350-6>
32. Z.H. Lin, Q.L. Wu, X.Q. Liu, H.Y. Ma, H. Liu et al., Flexible meta-tape with wide gamut, low lightness and low infrared emissivity for visible-infrared camouflage. Adv. Mater. **36**, 2410336 (2024). <https://doi.org/10.1002/adma.202410336>
33. M.Q. Zhang, P. Wang, X.H. Liu, Y.L. Fan, H.Y. Wang et al., Responsive metasurface for directional control of laser and thermal emission dynamic regulation. Adv. Mater. **37**, 2506061 (2025). <https://doi.org/10.1002/adma.202506061>
34. J. Luo, X. Fang, X. Liu, Z. Wu, Y.N. Zeng et al., Functional multispectral camouflage strategy based on flexible transparent metamaterial compatible with radiative cooling. Laser Photonics Rev. **19**, 2401905 (2025). <https://doi.org/10.1002/lpor.202401905>
35. C.Y. Liu, T. He, C.C. Hu, Q. Qian, Y.L. Hao et al., The Optimized design of sandwich structured SiO_2_/C@SiC/SiO_2_ composites through numerical simulation for temperature-resistant radar and infrared compatible stealth. Adv. Funct. Mater. **35**, 2416108 (2025). <https://doi.org/10.1002/adfm.202416108>
36. C.Y. Wen, B. Zhao, Y.H. Liu, C.Y. Xu, Y.Y. Wu et al., Flexible MXene-based composite films for multi-spectra defense in radar, infrared and visible light bands. Adv. Funct. Mater. **33**, 2214223 (2023). <https://doi.org/10.1002/adfm.202214223>
37. L. Chang, T.T. Liu, X.X. Fan, X.T. Zhang, X.C. Zhang et al., MXene derived aerogel with hetero-dimensional for multispectral response and devices. J. Mater. Sci. Technol. **245**, 77-87 (2026). <https://doi.org/10.1016/j.jmst.2025.05.026>
38. S. Zhang, F.S. Wu, F.Y. Hu, P.Y. Hu, M.P. Li et al., Cross-dimensional assembly of MXene/SiO_2_/KNF composite aerogels for radar and infrared stealth. Mater. Horiz. **12**, 6862-6874 (2025). <https://doi.org/10.1039/d5mh00667h>
39. T.T. Liu, Q. Zheng, W.Q. Cao, Y.Z. Wang, M. Zhang et al., In situ atomic reconstruction engineering modulating graphene-like MXene-based multifunctional electromagnetic devices covering multi-spectrum. Nano-Micro Lett. **16**, 173 (2024). <https://doi.org/10.1007/s40820-024-01391-8>
40. H. Zhu, Q. Li, C. Tao, Y. Hong, Z. Xu et al., Multispectral camouflage for infrared, visible, lasers and microwave with radiative cooling. Nat. Commun. **12**, 1805 (2021). <https://doi.org/10.1038/s41467-021-22051-0>
